# Pancreatic Cancer Health Disparity: Pharmacologic Anthropology

**DOI:** 10.3390/cancers15205070

**Published:** 2023-10-20

**Authors:** Nathan R. Wall, Ryan N. Fuller, Ann Morcos, Marino De Leon

**Affiliations:** 1 Division of Biochemistry, Department of Basic Science, Center for Health Disparities and Molecular Medicine, Loma Linda University, Loma Linda, CA 92350, USA; rnfuller@students.llu.edu (R.N.F.); amorcos@students.llu.edu (A.M.); 2 Division of Physiology, Department of Basic Science, Center for Health Disparities and Molecular Medicine, Loma Linda University, Loma Linda, CA 92350, USA; mdeleon@llu.edu

**Keywords:** pancreatic cancer, health disparities, pharmacologic anthropology, socio-cultural factors, patient care

## Abstract

**Simple Summary:**

Pancreatic cancer (PCa) is a highly aggressive and deadly form of cancer with a low five-year survival rate. This paper explores the role of pharmacologic anthropology in understanding and addressing health disparities related to PCa. Pharmacologic anthropology examines how cultural, social, economic, and behavioral factors affect the use and effectiveness of pharmaceutical treatments. In the context of PCa, it helps us understand why different population groups experience disparities in PCa outcomes. By adopting this interdisciplinary approach, researchers, healthcare providers, and policymakers can better understand the complex dynamics of PCa health disparities. This understanding can lead to culturally sensitive interventions, improved communication between patients and providers, increased community engagement, and policies that enhance access to quality care for all PCa patients.

**Abstract:**

Pancreatic cancer (PCa) remains a formidable global health challenge, with high mortality rates and limited treatment options. While advancements in pharmacology have led to improved outcomes for various cancers, PCa continues to exhibit significant health disparities, disproportionately affecting certain populations. This paper explores the intersection of pharmacology and anthropology in understanding the health disparities associated with PCa. By considering the socio-cultural, economic, and behavioral factors that influence the development, diagnosis, treatment, and outcomes of PCa, pharmacologic anthropology provides a comprehensive framework to address these disparities and improve patient care.

## 1. Introduction

Pancreatic cancer (PCa) is one of the most aggressive and lethal forms of cancer, with a five-year survival rate below 12% [1,2]. Despite advancements in cancer research and treatment, PCa outcomes remain poor, and significant disparities exist among different population groups [3,4,5,6]. This paper aims to examine the role pharmacologic anthropology plays in understanding and addressing the health disparities related to PCa.

Pharmacologic anthropology is an interdisciplinary field that explores the dynamic relationship between pharmacology (the study of drugs and their effects on the body) and anthropology (the study of human societies and cultures) [7]. It focuses on understanding the impact of cultural, social, economic, and behavioral factors on the use, efficacy, and outcomes of pharmaceutical interventions. Within the context of PCa, pharmacologic anthropology provides a framework to investigate the health disparities associated with this devastating disease.

PCa is characterized by its aggressive nature, late-stage diagnosis, limited treatment options, and poor survival rates [8,9]. However, the burden of PCa is not uniformly distributed among populations [10,11,12,13]. Health disparities related to PCa manifest in various ways, such as unequal access to healthcare, disparities in disease awareness and screening, differences in treatment outcomes, and varying rates of disease prevalence and mortality across different population groups [14,15].

Pharmacologic anthropology, as applied to PCa, seeks to understand the multifaceted nature of these health disparities. It recognizes that the development, diagnosis, treatment, and outcomes of PCa are influenced not only by biological factors but also by socio-cultural contexts and individual behaviors. By adopting an anthropological lens, this field acknowledges that healthcare practices, beliefs, and systems are embedded within specific cultural and social contexts and are influenced by factors such as race, ethnicity, socioeconomic status, and gender.

The scope of pharmacologic anthropology in the context of cancer in general and PCa specifically is broad and encompasses several aspects such as cultural and behavioral factors, socioeconomic factors, access to healthcare and treatment disparities, as well as genetic and biological factors. It investigates the impact of cultural norms and traditions on treatment-seeking behaviors [16], patient adherence to prescribed medications [17], and acceptance of alternative or complementary therapies [18,19]. This field explores the role of socioeconomic disparities in the prevention, diagnosis, and treatment of PCa. It considers how factors such as income, education, occupation, and access to healthcare facilities influence the availability and utilization of pharmacological interventions, leading to disparities in health outcomes. Pharmacologic anthropology investigates the barriers that limit access to appropriate healthcare services for PCa patients. It examines disparities in healthcare infrastructure, insurance coverage, geographic location, and healthcare provider bias, which can affect timely diagnosis, treatment initiation, and overall quality of care [20,21,22,23,24,25,26]. While pharmacologic anthropology primarily focuses on the social and cultural dimensions of health disparities, it also recognizes the importance of genetic and biological factors in PCa. It acknowledges the interplay between genetic predispositions, molecular pathways, and pharmacological responses, seeking to understand how these factors contribute to differential treatment outcomes among diverse populations [27,28,29,30,31].

By embracing these principles, researchers, healthcare providers, and policymakers can gain a more comprehensive understanding of the complex dynamics surrounding PCa health disparities. This interdisciplinary approach helps to identify and address the root causes of these disparities, inform culturally sensitive interventions, enhance patient-provider communication, promote community engagement, and advocate for policies that improve access to quality care for all individuals affected by PCa.

## 2. The Interplay between Pharmacology and Anthropology

PCa provides a compelling context to explore the interplay between pharmacology and anthropology. Understanding this interplay is crucial for comprehending the multifaceted factors that influence the development, treatment, and outcomes of PCa within diverse populations. Pharmacology, as the study of drugs and their effects, plays a central role in PCa treatment. It encompasses the discovery, development, and utilization of pharmaceutical interventions such as chemotherapy, targeted therapies, immunotherapies, and supportive medications. By analyzing the social and cultural contexts in which pharmaceutical interventions are utilized, anthropology sheds light on the complex interactions between individuals, communities, and healthcare systems.

The interplay between pharmacology and anthropology in the context of PCa can be observed in several ways: cultural perceptions and beliefs, treatment decision-making, socioeconomic disparities, patient-provider interactions, and health systems and policies. Anthropological healthcare research explores how cultural beliefs and perceptions regarding illness, cancer, and pharmaceutical interventions influence the acceptance and utilization of specific treatments for PCa [32,33]. Understanding cultural attitudes towards medications, side effects, and alternative therapies provides valuable insights for pharmacologists and healthcare providers, facilitating the development of patient-centered treatment approaches. Anthropology contributes to understanding how social and cultural factors impact treatment decision-making processes among individuals with PCa. Factors such as family dynamics, social support networks, religious or spiritual beliefs, and cultural expectations shape patients’ choices regarding the use of pharmacological interventions, participation in clinical trials, or engagement with complementary and alternative medicine. The interplay between pharmacology and anthropology also encompasses the examination of socioeconomic factors that contribute to health disparities in PCa [34,35,36,37,38]. Anthropological investigations explore how economic constraints, access to healthcare resources, insurance coverage, and social inequalities influence patients’ ability to access and afford potentially life-saving pharmacological treatments and shed light on the dynamics of patient-provider interactions and their influence on treatment outcomes. By understanding cultural norms of communication, healthcare-seeking behaviors, and expectations within specific communities, healthcare providers can engage in culturally sensitive and effective communication, leading to improved patient understanding, treatment adherence, and overall outcomes [39,40]. The interplay between pharmacology and anthropology extends to the examination of health systems and policies that shape the availability and accessibility of pharmacological interventions for PCa. Anthropological perspectives consider the structural, economic, and political factors that influence drug development, pricing, regulatory frameworks, and healthcare delivery, with the goal of advocating for equitable access to effective treatments [41,42,43].

By recognizing the interplay between pharmacology and anthropology, researchers, healthcare providers, and policymakers can develop a comprehensive understanding of the complex factors influencing PCa outcomes. This interdisciplinary approach allows for the development of patient-centered interventions, tailored healthcare strategies, and policies that address health disparities, enhance treatment efficacy, and improve the overall well-being of individuals affected by PCa.

## 3. Pancreatic Cancer Health Disparities

### 3.1. Socioeconomic Factors

Socioeconomic factors play a significant role in the occurrence, diagnosis, treatment, and outcomes of PCa, contributing to health disparities observed in different populations [44,45,46,47,48]. These factors encompass a range of social and economic conditions that influence access to healthcare, quality of care, and overall health outcomes. Understanding the impact of socioeconomic factors such as income and education, health insurance coverage, geographic location, occupation and work environment, and health literacy and health knowledge is crucial for addressing PCa health disparities effectively.

**Figure 1:**
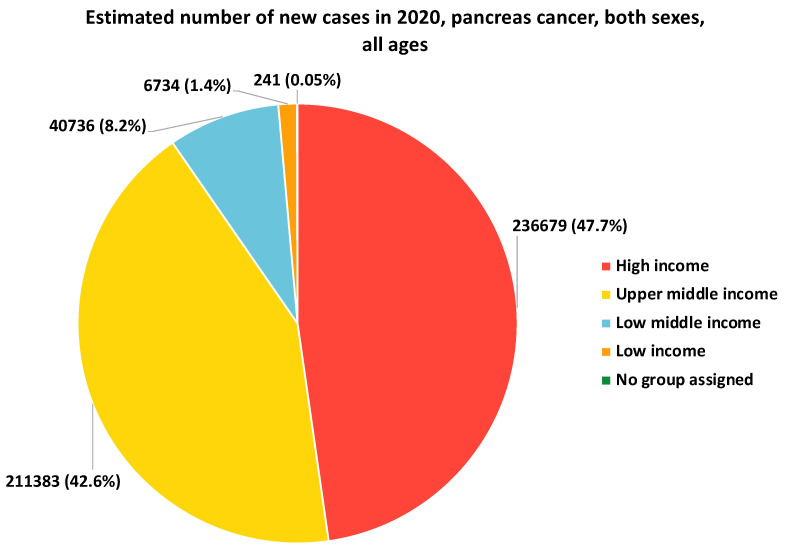
Estimated number of new cases in 2020 of pancreatic cancer, both sexes and all ages. Low-income countries generally have limited resources and healthcare infrastructure, which can impact cancer diagnosis and reporting. This is in contrast with high-income countries, where factors such as better screening, early detection, and improved treatment options contribute to the higher incidence reported. Data source: Adapted from the International Agency for Research on Cancer (IARC). GLOBOCAN 2020: Graph Production: Global Cancer Observatory (http://gco.iarc.fr/, accessed on 9 October 2023).

Lower income levels and limited educational attainment have been associated with an increased risk of PCa. However, the data would indicate that the estimated number of new cases of pancreatic cancer in low-income countries and individuals is often lower than in higher-income countries and individuals (Figure 1). Socioeconomic disadvantage can contribute to unhealthy lifestyle behaviors, such as tobacco use, poor diet, and limited access to preventive healthcare services. Individuals with lower income and education levels may also face barriers to early detection and timely access to appropriate treatment, resulting in poorer outcomes [49,50,51] (Figure 1).

Lack of health insurance or inadequate coverage is a significant socioeconomic factor contributing to PCa health disparities. Individuals without insurance or with limited coverage may delay seeking medical care, receive less comprehensive diagnostic evaluations, experience delays in treatment initiation, or have limited access to innovative therapies. Insurance status significantly influences the quality and timeliness of PCa care [21].

Geographic disparities play a role in PCa outcomes. Rural and underserved areas often have limited healthcare infrastructure, including oncology centers and specialized medical professionals [52,53]. Limited access to healthcare services, including cancer screening and treatment facilities, can result in delayed diagnosis, reduced treatment options, and poorer survival rates for individuals residing in these areas [54].

Certain occupations and occupational exposures have been associated with an increased risk of PCa. Occupational factors, such as exposure to carcinogens like asbestos, pesticides, or heavy metals, can contribute to the development of PCa [55,56,57]. Furthermore, job-related factors, including limited access to sick leave [58,59], occupational stress, and job insecurity, can impact healthcare-seeking behaviors and adherence to treatment among individuals diagnosed with PCa [60].

Socioeconomic disparities in health literacy and knowledge about PCa can influence individuals’ ability to understand and navigate the healthcare system effectively [61,62,63]. Limited health literacy can hinder comprehension of medical information, impede shared decision-making, and compromise adherence to treatment regimens. Individuals with low health literacy may have challenges understanding the importance of early detection, participating in clinical trials, or engaging in preventive behaviors [62,64].

Addressing socioeconomic factors in PCa health disparities requires a multi-faceted approach that includes improving access to healthcare, enhancing health education and awareness, supporting the socioeconomically disadvantaged, strengthening occupational health and safety, and enhancing health literacy. Efforts should focus on increasing access to affordable healthcare services, including cancer screenings, diagnostic tests, and specialized treatment centers. Expanding health insurance coverage, particularly for vulnerable populations, can reduce financial barriers and ensure timely access to necessary care. Implementing targeted health education campaigns can improve awareness about PCa risk factors, symptoms, and the importance of early detection [61]. Culturally tailored educational materials and outreach programs can help bridge knowledge gaps and promote preventive behaviors. Providing support services, such as patient navigation programs, transportation assistance, and financial counseling, can help mitigate the financial burden associated with cancer treatment and improve healthcare access for socioeconomically disadvantaged individuals [3,12,26,50]. Implementing and enforcing workplace safety regulations, promoting awareness of occupational hazards, and providing adequate protective measures can reduce occupational exposures that contribute to PCa risk. Developing and disseminating clear and accessible health information materials, using plain language, and ensuring effective communication between healthcare providers and patients can improve health literacy and empower individuals to make informed decisions about their PCa care. By addressing socioeconomic factors, healthcare systems can strive to reduce disparities in PCa outcomes, promote equity in access to quality care, and improve overall survival rates for all individuals affected by this devastating disease.

### 3.2. Cultural and Behavioral Factors

Cultural and behavioral factors significantly contribute to health disparities in PCa, shaping individuals’ beliefs, attitudes, and behaviors related to prevention, diagnosis, treatment-seeking, and adherence [65,66]. Understanding the cultural and behavioral influences on PCa can help identify strategies to address disparities and improve outcomes within diverse populations. Cultural beliefs and perceptions about cancer, including PCa, can impact health-seeking behaviors [67,68,69]. Cultural beliefs about the causes of cancer, treatment efficacy, and the role of traditional or alternative therapies may influence individuals’ decisions to seek medical care, adhere to treatment regimens, or participate in clinical trials. Understanding and addressing these beliefs are crucial for effective communication and patient engagement. Limited health literacy and language barriers can hinder understanding and engagement with PCa-related information and healthcare services. Cultural nuances and language barriers may impede effective communication between healthcare providers and patients, leading to misunderstandings, delayed diagnoses, or inadequate treatment. Providing culturally appropriate and linguistically accessible health information is essential to overcoming these barriers. Stigmas associated with cancer, including PCa, can affect individuals’ willingness to discuss symptoms, seek medical care, or disclose their diagnosis [70,71]. Cultural taboos, such as discussing health matters openly or seeking help outside the family or community, may hinder early detection and access to appropriate treatment. Efforts to reduce stigma, raise awareness, and create safe spaces for discussions about PCa are critical to overcoming these cultural barriers [72]. Traditional healing practices and complementary and alternative medicine play a significant role in some cultural communities. Integrating traditional healing practices with evidence-based medicine can help improve cultural acceptance and patient engagement [73,74,75]. Collaboration between healthcare providers and traditional healers can facilitate a more comprehensive approach to care. Cultural norms and familial decision-making dynamics can influence treatment decisions and adherence. In some cultures, decisions about healthcare may be made collectively within the family, with the patient’s autonomy potentially influenced by family members. Understanding these dynamics is crucial for effective communication, shared decision-making, and ensuring patient-centered care. Cultural factors, such as language barriers, immigration status, and discrimination, may limit access to healthcare services for certain population groups [76,77,78,79]. Mistrust of the healthcare system, particularly among marginalized communities, can lead to delayed diagnosis, treatment refusal, or suboptimal utilization of available resources. Building trust, promoting culturally competent care, and addressing healthcare disparities are essential to improving access for all individuals affected by PCa.

Addressing cultural and behavioral factors in PCa health disparities requires culturally sensitive strategies. These strategies may include culturally tailored education and outreach, community engagement, patient navigation and support, healthcare provider cultural competence, or a multidisciplinary collaboration [80,81,82]. Developing culturally appropriate educational materials and community outreach programs can help raise awareness, dispel myths, and promote early detection and screening for PCa within diverse populations. Collaborating with community leaders, organizations, and healthcare providers can help build trust, address cultural beliefs, and improve access to PCa information and services [83]. Healthcare providers should receive training in cultural competence to enhance communication, understanding, and engagement with patients from diverse cultural backgrounds. Engaging a diverse multidisciplinary range of healthcare professionals, including oncologists, primary care physicians, social workers, and interpreters, can facilitate culturally competent care, address cultural barriers, and improve outcomes for individuals with PCa [84,85]. By addressing cultural and behavioral factors, healthcare systems can strive to reduce disparities in PCa outcomes and ensure equitable access to quality care for all individuals, irrespective of cultural background or beliefs.

### 3.3. Access to Healthcare and Treatment Disparities

Access to healthcare and treatment disparities significantly contribute to PCa health disparities [86]. These disparities manifest in various ways, including unequal access to healthcare services, disparities in disease awareness and screening, differences in treatment outcomes, and varying rates of disease prevalence and mortality across different population groups. Understanding and addressing access-related disparities is crucial for improving PCa outcomes and promoting health equity [86]. Disparities in healthcare infrastructure, including the availability and distribution of medical facilities, cancer centers, and specialized healthcare providers, can contribute to access disparities in PCa [87]. Uneven geographic distribution of healthcare resources can result in limited access to timely diagnosis, treatment, and follow-up care, particularly in rural and underserved areas. Individuals without insurance or with high out-of-pocket costs may delay seeking medical attention, forego preventive screenings, or face financial hardships in accessing and adhering to recommended treatments. Disparities in PCa screening and early detection contribute to differences in outcomes. Limited access to screening programs, a lack of awareness about the importance of early detection, and inadequate utilization of screening services can result in late-stage diagnoses and reduced treatment options [88,89,90,91]. Difficulties in navigating the healthcare system can impede access to timely and appropriate care for PCa. Complex healthcare systems, a lack of information or guidance, and fragmented care coordination can lead to delays in diagnosis, treatment initiation, and follow-up care. Socioeconomic factors, such as low income, limited education, and unemployment, can create barriers to accessing healthcare for PCa [27,44]. Financial constraints may prevent individuals from seeking medical care, obtaining necessary diagnostic tests, or adhering to treatment plans. Racial and ethnic minorities often face higher rates of PCa incidence and poorer outcomes. Disparities in access to healthcare services, including culturally competent care, language barriers, and implicit biases, contribute to these disparities [13,62,70]. Addressing racial and ethnic disparities requires a focus on improving access, providing culturally sensitive care, and reducing healthcare provider biases.

Addressing access to healthcare and treatment disparities in PCa requires comprehensive strategies. Policy initiatives focused on expanding healthcare coverage, improving insurance affordability, and reducing barriers to access can help mitigate disparities. Advocacy efforts can raise awareness about PCa health disparities and push for policy changes that promote equity in access to care [92]. Community-based outreach and education programs can increase awareness about PCa, promote screening initiatives, and provide information about available resources and support services [61,68]. Culturally sensitive and linguistically appropriate materials can help overcome language and cultural barriers. Patient navigation programs and care coordination services can support individuals in navigating the healthcare system, facilitating timely diagnosis, treatment initiation, and follow-up care. Patient navigators can help address barriers, provide support, and connect patients to needed services. Healthcare providers should receive training in cultural competence to better understand and address the unique needs and challenges faced by diverse populations [21,41,42]. Culturally competent care ensures that individuals receive respectful, responsive, and effective healthcare services. Efforts should focus on reducing socioeconomic barriers to accessing healthcare, including expanding Medicaid programs, implementing sliding-scale fees, and providing financial assistance programs to ensure affordability of care for all individuals [93,94]. Continued research and data collection are crucial for understanding the specific factors contributing to access disparities in PCa. This knowledge can inform targeted interventions and policies to reduce disparities and improve outcomes. By addressing access to healthcare and treatment disparities, healthcare systems can strive to eliminate PCa health disparities, improve outcomes, and ensure equitable access to quality care for all individuals affected by the disease.

### 3.4. Genetic and Biological Factors

Genetic and biological factors play a significant role in PCa health disparities, contributing to variations in disease incidence, prognosis, and response to treatment among different population groups [95,96]. Understanding the genetic and biological factors associated with PCa can help identify potential risk factors, develop targeted interventions, and personalize treatment approaches to address disparities effectively. Certain genetic mutations and inherited syndromes have been identified as risk factors for PCa. For example, mutations in genes such as BRCA1, BRCA2, PALB2, and ATM are associated with an increased risk of developing PCa [97,98,99]. These genetic factors can vary across populations, contributing to differences in PCa incidence and prevalence among different ethnic groups. PCa exhibits heterogeneity in terms of tumor biology and molecular subtypes. Variations in tumor characteristics, such as tumor stage, histology, and molecular alterations, can impact treatment response and overall prognosis [100,101,102]. Understanding the biological factors underlying tumor heterogeneity can help develop tailored treatment strategies and identify novel therapeutic targets. Inflammation and metabolic dysregulation are implicated in PCa development and progression. Genetic and biological factors that influence chronic inflammation, such as variations in immune response genes, can contribute to disparities in PCa risk and outcomes. Additionally, metabolic conditions such as obesity and diabetes [103,104], which have genetic and biological underpinnings, are associated with an increased risk of PCa and may contribute to disparities. Genetic variations can impact drug metabolism, efficacy, and toxicity. Pharmacogenomic factors can influence individual responses to chemotherapy, targeted therapies, and other pharmacological interventions for PCa [105]. Variations in genes involved in drug metabolism and response pathways can contribute to disparities in treatment outcomes and side effects among different populations. The tumor microenvironment, including the immune system and stromal components, influences PCa progression and treatment response [88,90,98,106].

Understanding the genetic and biological factors contributing to PCa health disparities can inform strategies to address these disparities. Implementing genetic testing and counseling programs can help identify individuals at increased genetic risk for PCa, particularly those from high-risk ethnic groups or those with a family history. Offering targeted screening and preventive measures to individuals with identified genetic mutations can reduce disparities in PCa incidence and mortality [107,108,109]. Integrating genomic profiling and molecular characterization of tumors can help guide treatment decisions, identify potential therapeutic targets, and develop personalized treatment strategies. Tailoring treatment regimens based on individual genetic and biological factors can improve treatment efficacy and outcomes, bridging disparities among different populations [110,111]. Conducting research focused on understanding the genetic and biological factors that contribute to PCa disparities is essential, which is often referred to as precision medicine. By unraveling the molecular underpinnings of disparities, researchers can develop targeted interventions and therapies to address specific genetic and biological vulnerabilities [112,113,114]. Ensuring diversity and representation in genetic research studies is critical to addressing disparities effectively. Efforts should be made to include diverse populations in genetic research initiatives, considering factors such as ancestry, ethnicity, and genetic variations specific to different population groups [115,116]. Improving access to genetic testing, counseling, and precision medicine approaches is essential for reducing disparities. By considering the genetic and biological factors associated with PCa, healthcare systems can develop targeted interventions, improve treatment outcomes, and advance health equity by addressing disparities in the disease.

### 3.5. Pancreatic Cancer Screening

As has already been articulated, pancreatic cancer is known for its aggressive nature and high mortality rates [117]. It has also been shown to occur in more than one member of the same family but is not thought to be hereditary in origin. This familial form of cancer is typically recognized because multiple family members on one side of the family are diagnosed with the same cancer, and it has a pattern of being diagnosed late in life [118,119]. Importantly, even though familial pancreatic cancer clusters in one family, the cause of this disease does not seem to follow a hereditary pattern and does not seem to be caused by a change in one gene. Instead, there appear to be many influences: a combination of several gene mutations and factors such as diet and exercise, drinking alcohol, and tobacco use [119]. Early detection remains a critical factor in improving patient outcomes, but health disparities exist in the area of screening, particularly for individuals with a family history of the disease [99,120].

One of the primary barriers to adequate pancreatic cancer screening among individuals with familial risk factors is their socioeconomic status [45]. It has been consistently demonstrated that individuals from lower socioeconomic backgrounds are much less likely to undergo physician-recommended cancer screening, which can be attributed to factors such as healthcare service limitations [121], financial constraints, and even reduced health literacy [64,122]. In addition, lower-income individuals or families often go without adequate health insurance coverage, which in itself can be a deterrent to seeking screening for pancreatic cancer earlier [123]. Limited access to healthcare facilities and transportation challenges in underserved communities can further curtail efforts to identify and help at-risk individuals. For those without insurance, the cost of screening tests and follow-up procedures can be prohibitively high and thus lead to delayed or missed opportunities for early detection [124].

Racial and ethnic disparities also play a part in the underutilization of pancreatic cancer screening among individuals with familial risk factors [125]. In the United States, African Americans and Hispanics are more likely to be diagnosed with pancreatic cancer at advanced stages, resulting in poorer outcomes [12,126]. This is partly attributed to disparities in healthcare access, socio-economic status, and a lack of awareness about the importance of early screening in these communities.

Healthcare provider disparities are another aspect of the problem [61]. Not all healthcare professionals are equally aware of the need for early screening of individuals with familial risk factors. This can lead to delayed or missed opportunities for patients to receive appropriate screening recommendations, particularly if their family history is not adequately documented or recognized.

In order to address pancreatic cancer screening disparities among individuals with familial risk factors, a multifaceted approach is needed. Public health campaigns and educational programs [127,128] should be developed to raise awareness about the importance of early screening, and these campaigns should work to identify those at risk and emphasize the benefits of screening and early detection. Expanding access to healthcare services and reducing financial barriers is crucial to reducing this health disparity. This may involve policies to increase government-provided health insurance coverage for those identified as most at risk [129]. The establishment of mobile screening clinics in underserved areas and programs to provide financial assistance and transportation assistance are also recommended [130]. Healthcare providers should receive cultural competency training to better serve diverse patient populations. This includes recognizing the significance of family history, but also that many may not know this history for longer than a couple generations [131]. Lastly, continued research into the genetic and environmental factors that contribute to familial pancreatic cancer is essential, as are the groups that advocate for changes in policy and funding support for innovative methods for early detection and interventions.

## 4. Pharmacologic Anthropology and Pancreatic Cancer

### 4.1. Understanding Local Knowledge and Practices

**Figure 2:**
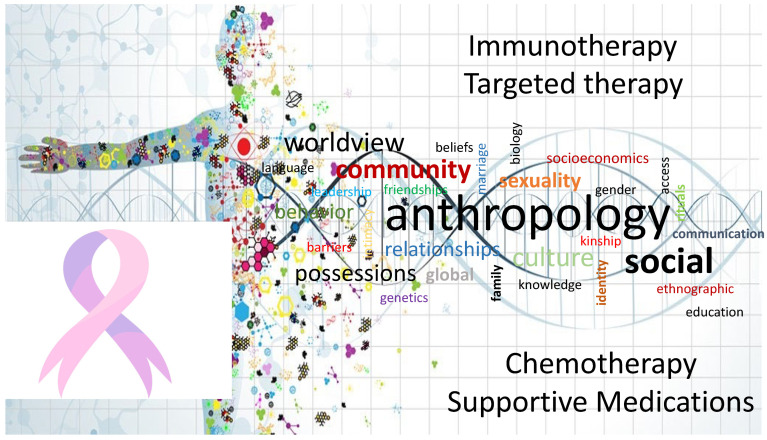
Understanding Health Disparities in Pancreatic Cancer through Pharmacologic Anthropology. In this illustrative representation, we depict the multifaceted characteristics of pharmacologic anthropology as it pertains to pancreatic cancer health disparities. The fusion of anthropological insights with pharmacological interventions is central to achieving more equitable outcomes in this challenging disease. By understanding cultural factors and community perspectives, we can tailor immunotherapies to be more effective and accessible to diverse populations. Through community engagement and culturally sensitive research, we identify genetic variations and tailor targeted therapies for better outcomes, and through culturally tailored approaches, the impact of traditional practices and beliefs on chemotherapy adherence can enhance its effectiveness. Supportive medications, including pain management and symptom control, are crucial in improving the quality of life for pancreatic cancer patients. A pharmacologic anthropology lens takes into account patients’ cultural preferences and beliefs to provide holistic and culturally sensitive support.

Pharmacologic anthropology explores the intersection between culture, society, and medicine, focusing on how local knowledge and practices influence health, healing, and the use of medications (Figure 2). In the context of PCa, understanding local knowledge and practices can provide valuable insights into cultural beliefs, traditional healing systems, and the use of pharmacological interventions. This understanding is crucial for addressing health disparities, promoting effective communication, and developing culturally appropriate strategies for prevention, treatment, and support. Local knowledge and cultural beliefs about PCa can shape individuals’ understanding of the disease, its causes, and available treatment options [132]. These beliefs may influence help-seeking behaviors, treatment preferences, and adherence to medical recommendations [133,134]. By acknowledging and respecting cultural beliefs, healthcare providers can build trust, enhance communication, and promote culturally sensitive care. Many cultures have traditional healing systems that coexist with biomedical approaches. These systems often involve the use of herbal remedies, dietary practices, spiritual healing, and other traditional interventions [135,136,137,138,139]. Understanding local healing practices and integrating them with evidence-based medicine [140,141,142] can help bridge the gap between traditional and biomedical approaches, improving patient acceptance and engagement with treatment. Local knowledge and practices can impact medication use and adherence among individuals with PCa. Cultural factors such as religious beliefs [143], taboos, and social norms may influence attitudes towards medications and treatment regimens. Understanding these factors can help healthcare providers develop tailored approaches to medication management and adherence support [144,145,146,147]. Local knowledge and practices influence health-seeking behavior and treatment decision-making processes. Factors such as family dynamics, community support, and trust in healthcare systems play significant roles in treatment decisions [148]. By understanding these factors, healthcare providers can better support individuals in making informed decisions and engaging in treatment options that align with their cultural values. Language and communication styles vary across cultures, impacting healthcare interactions. Effective communication requires cultural competence and sensitivity to overcome language barriers, ensure a clear understanding of medical information, and facilitate shared decision-making [149]. Utilizing interpreters, providing translated materials, and employing culturally appropriate communication strategies can enhance patient-provider communication [67,122]. Engaging local communities, community leaders, and traditional healers fosters trust and facilitates collaboration in addressing PCa. Working together with community stakeholders helps identify community-specific needs, build awareness, and develop culturally appropriate interventions that are embedded within the community’s social fabric [43,147,150,151].

To leverage the insights gained from understanding local knowledge and practices, several strategies can be implemented. Conducting community-based research allows for the inclusion of local knowledge and perspectives in PCa studies [43,82]. Engaging community members as active participants and co-researchers promotes mutual learning, increases community trust, and enhances the relevance and effectiveness of research findings [152,153]. Healthcare providers should receive training in cultural competence to develop an understanding of diverse cultural beliefs, practices, and communication styles [79]. This training improves healthcare providers’ ability to effectively engage with patients, respect cultural diversity, and integrate local knowledge into clinical practice [52,81,143]. Collaborating with traditional healers and integrating their knowledge and practices within the healthcare system can improve patient care. This collaboration allows for the exchange of information, mutual referrals, and coordinated care, ensuring a holistic approach to PCa management [147,150,151]. Developing culturally sensitive educational materials [154], awareness campaigns [69], and public health initiatives raises awareness about PCa within specific communities [136]. These materials should be adapted to the cultural and linguistic preferences of the target population, addressing local knowledge and practices. Adopting a patient-centered approach that values and incorporates the preferences, beliefs, and practices of individuals can improve treatment outcomes and patient satisfaction [155]. Recognizing and respecting patients’ cultural perspectives enhances patient-provider relationships, promotes trust, and facilitates shared decision-making [156,157]. By understanding local knowledge and practices, pharmacologic anthropology can contribute to reducing health disparities in PCa by incorporating cultural beliefs, leveraging traditional healing systems, and promoting culturally appropriate and patient-centered care.

### 4.2. Assessing Cultural Beliefs and Perceptions

Cultural beliefs and perceptions play a significant role in shaping individuals’ understanding of the disease, influencing health-seeking behaviors, treatment decisions, and medication adherence. By assessing and understanding these cultural beliefs, healthcare providers and researchers can develop culturally sensitive approaches to PCa prevention, treatment, and support, thereby addressing health disparities. Qualitative research methods, such as interviews, focus groups, and ethnographic observations, can be employed to explore cultural beliefs and perceptions related to PCa. These methods allow researchers to engage with individuals and communities, gaining insights into their perspectives, experiences, and cultural frameworks regarding the disease [158,159]. By applying a cultural lens, researchers can identify the cultural frameworks through which individuals understand and interpret PCa. This includes examining cultural values, beliefs, religious or spiritual practices, and social norms that influence perceptions of the disease [32,82,160]. By understanding these frameworks, healthcare providers can better tailor their approaches to meet patients’ cultural needs. Ethnomedicine refers to the traditional medical practices, beliefs, and knowledge systems that exist within a specific cultural group. Exploring ethnomedicine and traditional healing systems related to PCa can provide insights into alternative perspectives and treatment approaches that individuals may utilize alongside or instead of biomedical interventions [161,162,163]. Understanding the role of traditional healing systems allows for respectful collaboration and integration with biomedical care. Cultural beliefs and perceptions shape health-seeking behaviors and practices [164]. Assessing how cultural beliefs influence individuals’ decisions to seek medical care, their perceptions of symptoms, and their preferences for treatment can shed light on disparities in access and utilization of healthcare services [62,82]. Understanding these behaviors helps healthcare providers tailor interventions to effectively engage and support individuals at risk of or affected by PCa. Cultural beliefs and perceptions may contribute to the stigma and taboos surrounding PCa. Assessing the cultural factors that contribute to stigma allows healthcare providers to address misconceptions, educate communities, and develop supportive interventions that reduce the burden of stigma and promote early detection and care-seeking behaviors [52,67,71]. Effective health communication requires an understanding of cultural beliefs and perceptions. Assessing cultural beliefs helps tailor health messages and educational materials to be culturally appropriate, linguistically accessible, and respectful of diverse cultural perspectives [81,156]. This promotes better understanding, acceptance, and engagement with PCa prevention and treatment efforts.

By assessing cultural beliefs and perceptions related to PCa, pharmacologic anthropology provides valuable insights for addressing health disparities. Findings from cultural assessments can inform the development of culturally sensitive educational materials and awareness campaigns [81,156]. These initiatives can dispel myths, address cultural misconceptions, and increase knowledge about PCa within specific cultural groups. Understanding cultural beliefs and perceptions allows for the design of interventions that align with the cultural frameworks of specific populations [67,71,153]. This includes incorporating traditional healing practices, addressing cultural values, and considering culturally acceptable approaches to prevention, treatment, and support. Assessing cultural beliefs and perceptions fosters community engagement by involving community members as active participants in addressing PCa disparities. Engaging community leaders, organizations, and cultural influencers helps ensure that interventions are well-received, culturally relevant, and aligned with community needs [165,166,167]. Awareness of cultural beliefs and perceptions improves patient-provider communication [168,169]. Healthcare providers can engage in open and respectful discussions, acknowledge cultural perspectives, and incorporate patients’ beliefs into shared decision-making processes. Assessing cultural beliefs and perceptions related to PCa through the lens of pharmacologic anthropology enhances our understanding of the cultural factors that shape health behaviors, treatment choices, and health outcomes.

### 4.3. Community Engagement and Health Education

Pharmacologic anthropology emphasizes the importance of community engagement and health education in addressing PCa disparities. By actively involving communities in the development and implementation of interventions and by providing culturally appropriate health education, healthcare providers can empower individuals and communities to make informed decisions about PCa prevention, treatment, and support. Community-Based Participatory Research (CBPR) involves collaborating with community members, organizations, and stakeholders in all phases of the research process. Engaging the community as equal partners fosters mutual learning, builds trust, and ensures that interventions are grounded in the community’s needs, values, and cultural contexts. This approach promotes community ownership and the sustainability of interventions [170,171,172,173]. Cultural competence in health education involves understanding and respecting cultural beliefs, practices, and communication styles. Healthcare providers should develop educational materials that are culturally appropriate, linguistically accessible, and visually appealing [174,175]. Taking into account literacy levels, language preferences, and cultural sensitivities ensures that health education materials effectively reach and engage diverse populations [62,122]. Developing tailored health education programs specific to PCa can address the unique needs and challenges of different communities. These programs should incorporate cultural beliefs, cultural norms, and local contexts into the content and delivery methods [61,63,149]. Utilizing community health workers, cultural ambassadors, or trusted individuals from the community can enhance the effectiveness of education efforts. Health literacy plays a crucial role in understanding health information and making informed decisions [62,122]. Health education initiatives should focus on improving health literacy by using plain language, visual aids, and interactive formats. It is important to ensure that health information is accessible to individuals with varying levels of education and health literacy. Engaging in community outreach activities and awareness campaigns raises awareness about PCa, its risk factors and symptoms, and the importance of early detection. These initiatives can include health fairs, community events, media campaigns, and partnerships with local organizations [152]. Culturally appropriate messaging and outreach strategies can maximize community participation and engagement. Health education should empower individuals and communities to become advocates for their health. This can include providing information on screening guidelines, risk reduction strategies, and resources for accessing healthcare services [176,177,178]. Encouraging individuals to ask questions, seek second opinions, and actively participate in their care can lead to improved health outcomes and reduced disparities. Creating support groups and peer networks for individuals and families affected by PCa can provide emotional support, shared experiences, and practical guidance. These groups can be tailored to specific cultural groups, ensuring a safe space for discussing cultural concerns, addressing language barriers, and sharing culturally relevant coping strategies [179,180,181,182,183]. Community health workers (CHWs) play a vital role in bridging gaps in healthcare access and communication. Training CHWs in PCa awareness, prevention, and support equips them to serve as trusted sources of information within their communities [39,43]. CHWs can provide culturally sensitive education, navigate healthcare systems, and connect individuals to appropriate resources. By actively engaging communities and providing culturally appropriate health education, pharmacologic anthropology contributes to reducing PCa disparities. These approaches promote community empowerment, enhance health literacy, and foster partnerships that are essential for improving PCa prevention, treatment, and support within diverse populations.

### 4.4. Addressing Structural and Systemic Barriers

Pharmacologic anthropology recognizes the importance of addressing structural and systemic barriers in order to reduce PCa disparities. These barriers can disproportionately affect certain populations, including marginalized communities, low-income individuals, and racial/ethnic minorities. By understanding and addressing these barriers, healthcare providers and policymakers can work towards creating a more equitable healthcare system for PCa prevention, diagnosis, treatment, and support. Structural barriers, such as lack of health insurance, limited healthcare facilities, transportation challenges, and long waiting times, can hinder access to timely and quality healthcare services [184]. Efforts should be made to expand access to affordable healthcare, improve the geographic distribution of healthcare facilities, and provide transportation options for individuals to access PCa screenings, diagnostic tests, treatments, and follow-up care. Disparities in health insurance coverage contribute to inequitable access to PCa care. Efforts should be directed towards expanding healthcare coverage and reducing insurance barriers, particularly for low-income individuals and underserved populations [44,53,76]. This includes promoting Medicaid expansion, implementing health insurance programs for uninsured individuals, and advocating for policies that ensure comprehensive coverage for PCa-related services [76,167]. Socioeconomic factors, such as income, education, employment, and housing, can impact an individual’s ability to access and afford healthcare. Addressing socioeconomic disparities requires a multifaceted approach, including policies that promote income equality, improve educational opportunities, create job stability, and provide affordable housing [45,50,167]. These efforts can help mitigate the impact of socioeconomic barriers on PCa outcomes. Language and cultural barriers can impede effective communication and understanding between healthcare providers and patients. Addressing language and cultural competence involves providing interpreter services, offering language-appropriate educational materials, and training healthcare providers in culturally sensitive care [122,147,184]. By ensuring effective communication, individuals from diverse cultural and linguistic backgrounds can better navigate the healthcare system and receive appropriate PCa care. Addressing disparities in research and funding is essential for advancing knowledge about PCa and developing targeted interventions. Efforts should be made to increase the representation of underrepresented populations in research studies, prioritize funding for research focused on health disparities, and promote collaborations between researchers and communities affected by PCa [92,162]. Policy changes and advocacy efforts can play a crucial role in addressing structural and systemic barriers [41,148]. Advocating for policies that promote health equity, increase access to care, and reduce healthcare disparities can create a more supportive environment for individuals with PCa [176]. This includes policies that support early detection and screening programs, eliminate discriminatory practices, and promote equity in healthcare delivery. Engaging communities affected by PCa in decision-making processes, program planning, and policy development is essential [170,178]. By involving community members, healthcare providers, researchers, and policymakers can gain insights into the unique challenges faced by different populations and develop solutions that address their specific needs. Community engagement fosters collaboration, builds trust, and ensures that interventions and policies are responsive to the concerns and priorities of those most affected by PCa disparities. Addressing structural and systemic barriers requires a comprehensive and multi-dimensional approach that involves collaboration among healthcare providers, researchers, policymakers, community organizations, and affected individuals. By acknowledging and working towards dismantling these barriers, pharmacologic anthropology contributes to creating a more equitable healthcare system for PCa prevention, diagnosis, treatment, and support.

### 4.5. Enhancing Patient-Provider Communication

Pharmacologic anthropology recognizes the importance of enhancing patient-provider communication in the context of PCa care. Effective communication is vital for building trust, promoting shared decision-making, ensuring patient understanding, and addressing the unique cultural and social factors that influence patients’ experiences and perspectives. Here are some ways in which pharmacologic anthropology can contribute to enhancing patient-provider communication. Healthcare providers should be aware of and respect patients’ cultural beliefs, values, and practices [185,186]. By acknowledging and understanding cultural differences, providers can establish rapport, promote trust, and create a safe and non-judgmental environment for open communication. Language barriers can impede effective communication. Healthcare providers should ensure access to professional interpretation services, including in-person interpreters or telephonic interpretation services, to facilitate communication with patients who have limited English proficiency [122,169,185]. This ensures that patients can express their concerns, understand medical information, and actively participate in their care. Healthcare providers should use plain language and avoid medical jargon when communicating with patients. They should assess patients’ health literacy levels and tailor their explanations and instructions accordingly [62,122,179]. Visual aids, written materials, and multimedia resources can also enhance patient understanding [61,63]. Healthcare providers should practice active listening, demonstrating genuine interest in patients’ concerns, and validating their experiences. This involves giving patients ample time to express their thoughts, asking open-ended questions, and using reflective techniques to ensure accurate understanding [187,188]. Active listening builds rapport, demonstrates empathy, and fosters a collaborative patient-provider relationship [176]. Healthcare providers need to promote patient-centered care, which involves recognizing and incorporating patients’ preferences, values, and goals into treatment decisions [189,190,191]. Healthcare providers should engage in shared decision-making by presenting treatment options, discussing risks and benefits, and considering patients’ individual circumstances and values [192,193]. This approach empowers patients to actively participate in their care and fosters a sense of ownership and autonomy. Different cultures have unique communication styles and preferences. Pharmacologic anthropology emphasizes the need for healthcare providers to adapt their communication approaches to align with patients’ cultural backgrounds. This may involve understanding cultural norms around deference to authority, family involvement in healthcare decisions, and the importance of non-verbal cues [194,195,196,197]. By adapting communication styles, healthcare providers can bridge cultural gaps and foster effective communication. Healthcare providers should provide clear and comprehensive information about PCa, its treatment options, potential side effects, and available support services [61,179]. They should use patient-friendly educational materials, visual aids, and multimedia resources to enhance patient comprehension [63,161]. Effective communication includes addressing patients’ emotional and psychosocial needs [159,191]. Healthcare providers should create a supportive environment where patients feel comfortable expressing their emotions and fears. Offering emotional support, empathy, and referrals to counseling or support groups can help patients cope with the emotional challenges of PCa. By integrating these principles into patient-provider communication, pharmacologic anthropology enhances understanding, trust, and collaboration between patients and healthcare providers in the context of PCa care. This promotes patient satisfaction, adherence to treatment plans, and improved health outcomes.

## 5. Case Studies and Best Practices

### 5.1. Successful Interventions in Addressing Health Disparities

**Figure 3:**
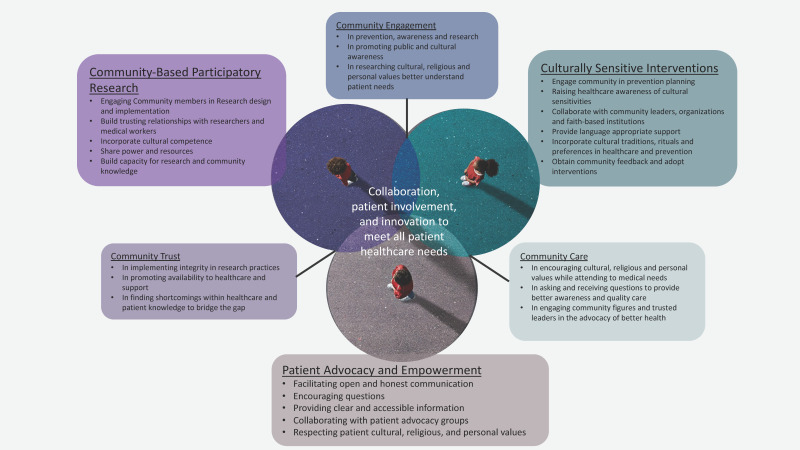
Case Studies and Best Practices: Case studies and best practices exemplify the principles of pharmacologic anthropology—focusing on culturally sensitive interventions; community-based participatory research; and patient advocacy and empowerment. These vital elements support collaboration and community engagement, fostering equitable healthcare outcomes.

While PCa remains a challenging disease with significant health disparities, several successful interventions have been implemented to address these disparities (Table 1). Here are a few case studies and best practices that have shown promise in reducing health disparities associated with PCa. Community-based interventions have proven effective in raising awareness, promoting early detection, and increasing access to care [198]. For example, the American Association for Cancer Research (AACR) and the Pancreatic Cancer Action Network (PanCAN) have implemented community outreach programs targeting African American populations, who are disproportionately affected by PCa. These programs provide culturally tailored education, screening events, and support services to increase awareness and promote early detection. Patient navigation programs aim to guide patients through the complexities of the healthcare system, providing support and assistance in accessing timely and appropriate care [199,200]. These programs have shown success in improving access to treatment, reducing delays in care, and enhancing patient satisfaction. For example, the Patient Navigation in Medically Underserved Areas (PNMUA) programs implemented by the American Cancer Society and the National Institutes of Health have demonstrated positive outcomes in reducing disparities among underserved populations, including those with limited access to healthcare resources [201,202]. Tailoring interventions to specific cultural groups can improve engagement, trust, and outcomes. For instance, the Asian Liver Center (ALC) at Stanford University developed a culturally tailored program called “Screening in the Community” to increase PCa screening among Asian populations. The program incorporates bilingual outreach, culturally appropriate educational materials, and community partnerships to address cultural barriers and increase participation in screening initiatives. Implementing targeted screening programs for high-risk populations has the potential to detect PCa at earlier stages, when treatment options are more effective. For example, the High-Risk Pancreatic Cancer Clinic (HRPCC) in the Skip Viragh Center for Pancreatic Cancer and the Sidney Kimmel Comprehensive Cancer Center at Johns Hopkins Medicine focus on screening individuals with a family history of PCa or known genetic mutations associated with the disease. This specialized clinic provides comprehensive risk assessment, genetic counseling, and surveillance strategies to identify PCa early in high-risk individuals. Health system changes, such as implementing multidisciplinary care teams, standardized treatment protocols, and quality improvement initiatives, can help reduce disparities in PCa outcomes. Institutions like MD Anderson Cancer Center and Gastrointestinal Center have established multidisciplinary clinics dedicated to PCa care, enabling coordinated and comprehensive treatment approaches that improve patient outcomes. Policy changes and advocacy play a crucial role in addressing disparities in PCa. For example, the Pancreatic Cancer Action Network has advocated for increased federal funding for PCa research, improved insurance coverage for treatments, and policies promoting early detection efforts. These initiatives aim to address structural barriers and promote equitable access to care and resources. Collaborative partnerships between healthcare providers, researchers, community organizations, and advocacy groups are essential in developing and implementing successful interventions (Figure 3). These partnerships leverage the expertise and resources of various stakeholders, ensuring that interventions are evidence-based, culturally sensitive, and tailored to the needs of specific populations. It is important to note that PCa health disparities are complex, and addressing them requires a multi-faceted and interdisciplinary approach. Continued research, evaluation of interventions, and collaboration among stakeholders are critical to identifying and implementing effective strategies to reduce disparities and improve outcomes for individuals affected by PCa.

### 5.2. Community-Based Participatory Research (CBPR)

CBPR is a collaborative research approach that involves the active engagement of community members, organizations, and researchers in all stages of this research process. CBPR has been successfully employed in PCa research, contributing to improved understanding of the disease, addressing health disparities, and developing effective interventions. Here are a few case studies and best practices showcasing the impact of CBPR in PCa. The Detroit Urban Research Center (DURC) is a partnership between community organizations, academic institutions, and researchers aimed at addressing health disparities in Detroit, including PCa. Through CBPR, the DURC engaged community members in the design and implementation of studies, community outreach programs, and policy advocacy efforts [203,204]. This collaborative approach facilitated community-driven research priorities, increased community involvement in research, and resulted in culturally tailored interventions for PCa prevention and treatment. CBPR has been used to study and address PCa disparities in Latino communities. Researchers collaborated with community organizations, health clinics, and individuals from Latino backgrounds to identify cultural beliefs, barriers to care, and information needs related to PCa [126,205]. The findings from these studies informed the development of culturally appropriate educational materials, community outreach programs, and navigation services to improve access to PCa prevention and treatment services among Latino populations [209]. The Appalachian Community Cancer Network (ACCN) is a collaborative initiative that employs CBPR to address cancer disparities in the Appalachian region of the United States, where PCa incidence and mortality rates are high. The ACCN engages community members, healthcare providers, and researchers to identify community needs, develop interventions, and evaluate their effectiveness. Through CBPR, the ACCN has implemented community education programs, screening initiatives, and survivorship support services to reduce PCa disparities in the Appalachian region [210]. CBPR has been instrumental in the development of survivorship programs for individuals living with PCa. Community members and survivors actively participated in the design and implementation of support services, including peer support groups, survivorship education workshops, and access to resources [206,207,208]. These programs address the unique physical, emotional, and psychosocial needs of PCa survivors, resulting in improved quality of life and enhanced survivorship outcomes.

Best practices in CBPR for PCa research include:

- Engaging community members from the outset: Involving community members in this research design, implementation, and dissemination phases ensures that research is responsive to community needs and priorities.
- Building trusting relationships: Establishing trust between researchers and community partners is crucial for successful CBPR. Transparent communication, mutual respect, and recognition of the expertise and contributions of all stakeholders help foster trust and collaboration.
- Incorporating cultural competence: Recognizing and addressing cultural factors that influence health beliefs, practices, and access to care is essential. Cultural competence training for researchers and incorporating cultural perspectives into research protocols ensure that interventions are culturally appropriate.
- Sharing power and resources: CBPR requires equitable power sharing and resource distribution among community partners and researchers. Meaningful involvement of community members in decision-making processes, capacity building, and resource allocation fosters a sense of ownership and sustainability.
- Capacity building: CBPR should include capacity-building efforts to enhance this research skills and knowledge of community members. This empowers community partners to actively contribute to the research process, strengthen their own advocacy efforts, and sustain interventions beyond this research project.

CBPR in PCa research has demonstrated its effectiveness in addressing health disparities, promoting community engagement, and developing interventions that are responsive to community needs. By leveraging the expertise of both researchers and community members, CBPR will contribute to more equitable and impactful approaches to PCa prevention, treatment, and support.

### 5.3. Culturally Sensitive Interventions

Culturally sensitive interventions are critical in addressing PCa disparities and ensuring that healthcare approaches are tailored to the cultural beliefs, values, and practices of diverse populations. Here we describe some case studies and best practices that highlight the importance of culturally sensitive interventions in PCa:

Project ECHO (Extension for Community Healthcare Outcomes) is a telehealth-based collaborative model that has been used at Rutgers Cancer Institute of New Jersey, MD Anderson Cancer Center, the National Institutes of Health, Indiana University at Purdue, and others to improve PCa care in underserved communities. Through this program, specialists connect with primary care providers and community health workers via videoconferencing to deliver training, mentorship, and support. Culturally sensitive approaches are incorporated by considering the unique cultural perspectives, health beliefs, and social determinants of health within each community. By leveraging local knowledge and understanding cultural contexts, Project ECHO promotes better communication and enhances the delivery of culturally sensitive care.

Native patient navigation programs have been implemented to improve access to PCa care among Indigenous communities. These programs employ patient navigators who are embedded within the community and possess cultural competency. Navigators provide support, education, and assistance in navigating the healthcare system, addressing cultural barriers, and promoting adherence to treatment. By understanding and respecting cultural practices, traditions, and beliefs, patient navigators can build trust and foster effective communication, resulting in improved outcomes for Native patients with PCa [211,212,213,214].

Faith-based organizations have played a significant role in implementing culturally sensitive interventions for PCa. For example, partnerships between healthcare providers and faith-based organizations have facilitated community outreach, education, and support programs [215,216,217,218]. These programs often incorporate faith-based messaging, utilize religious leaders as trusted messengers, and address spiritual needs and coping strategies specific to the cultural context of the community. By aligning interventions with religious and cultural values, faith-based partnerships enhance engagement and promote access to care.

Developing culturally tailored educational materials is essential to effectively communicating information about PCa prevention, risk factors, and early detection. This includes using appropriate language, visuals, and examples that resonate with the target population [219,220]. For instance, creating educational materials in multiple languages, using culturally relevant images, and considering health literacy levels can improve understanding and engagement among diverse communities. Collaborating with community members, cultural organizations, and healthcare providers ensures that the materials are contextually appropriate and sensitive to cultural nuances.

Community health workers (CHWs) have been instrumental in delivering culturally sensitive interventions for PCa. CHWs are members of the community who have a deep understanding of cultural norms, beliefs, and healthcare challenges. They provide education, support, and navigation services within their communities, bridging the gap between healthcare providers and community members [221,222,223]. By having culturally similar backgrounds, CHWs can effectively communicate and connect with individuals, addressing cultural barriers and tailoring interventions to specific cultural needs.

Best practices for culturally sensitive interventions in PCa include:

- Engage community members and stakeholders from the target population in the intervention planning, design, and implementation process.
- Conduct a thorough assessment of the cultural beliefs, values, practices, and health disparities prevalent within the community.
- Collaborate with community leaders, cultural organizations, and faith-based institutions to leverage existing resources and networks.
- Train healthcare providers and researchers in cultural competence to enhance their understanding of diverse populations and improve patient-provider communication.
- Provide language-appropriate and culturally relevant educational materials, ensuring they are easily understandable and accessible to the target population.
- Incorporate cultural traditions, rituals, and preferences into interventions to enhance engagement and acceptance.
- Evaluate and adapt interventions based on community feedback and outcomes to ensure ongoing cultural relevance and effectiveness.

By embracing culturally sensitive interventions, healthcare providers and researchers can improve access to care, enhance communication, and reduce disparities in PCa outcomes among diverse populations.

### 5.4. Patient Advocacy and Empowerment

Patient advocacy and empowerment play a crucial role in addressing PCa disparities by ensuring that patients have a voice, are actively involved in their care decisions, and have access to the resources and support they need. Here are some case studies and best practices that highlight the importance of patient advocacy and empowerment in PCa:

PanCAN is a patient advocacy organization dedicated to improving the lives of individuals affected by PCa. They provide resources, support, and advocacy opportunities for patients and their families. PanCAN has been successful in raising awareness about PCa, advocating for increased research funding, and promoting policies that improve access to quality care. Through their advocacy efforts, they empower patients and their families to become informed advocates for themselves and others.

Support groups for PCa patients and caregivers provide a platform for sharing experiences, knowledge, and emotional support. These groups empower patients by fostering a sense of community, reducing isolation, and providing opportunities to learn from others who have faced similar challenges. Support groups often collaborate with healthcare providers to ensure that patients receive accurate information and access to resources, enhancing patient empowerment [124,224].

Shared decision-making involves healthcare providers and patients working together to make informed treatment decisions based on the patient’s values, preferences, and available evidence [225,226]. In PCa care, this approach ensures that patients are actively involved in their treatment plans, understand the risks and benefits of different options, and have their values and goals considered. By empowering patients to participate in decision-making, shared decision-making enhances patient autonomy and promotes patient-centered care.

Providing comprehensive and understandable health education materials empowers patients to actively engage in their care. Educational resources should cover various aspects of PCa, including treatment options, potential side effects, symptom management, and survivorship [227,228,229]. Accessible information enables patients to make informed decisions, ask relevant questions, and better navigate their cancer journey.

Empowering patients does not end with treatment completion. Survivorship programs for PCa patients focus on addressing physical, emotional, and psychosocial needs after treatment. These programs offer resources, support, and survivorship care plans to empower patients to manage their long-term health, cope with potential side effects, and promote overall well-being [179,222]. Survivorship programs often include educational workshops, counseling services, and connections to support networks.

Patient navigation programs assist patients in navigating the healthcare system, coordinating care, and accessing necessary resources. Patient navigators act as advocates, providing support, guidance, and information throughout the cancer journey. By empowering patients with knowledge and assistance, patient navigation programs help overcome barriers to care, improve health outcomes, and enhance patient empowerment [108,152].

Online communities and forums provide a platform for patients and caregivers to connect, share information, and seek support [69,152]. These communities empower patients by enabling them to learn from others’ experiences, access up-to-date information, and find emotional support from individuals who understand their challenges. Patient communities foster a sense of empowerment through shared knowledge and shared decision-making.

Best practices for patient advocacy and empowerment in PCa include:

- Facilitating open and honest communication between patients and healthcare providers ensures that patients are active participants in their care.
- Encouraging patients to ask questions, seek second opinions, and actively engage in treatment decision-making.
- Providing clear and accessible information about treatment options, clinical trials, and supportive care services.
- Collaborating with patient advocacy organizations to connect patients with resources, support groups, and educational materials.
- Promoting patient education and health literacy to enhance patients’ understanding of their diagnosis, treatment, and self-care.
- Advocating for policies that improve access to affordable healthcare, clinical trials, and supportive services for PCa patients.
- Recognizing and respecting patients’ cultural, religious, and personal values when developing care plans and support services.

By prioritizing patient advocacy and empowerment in PCa care, healthcare providers, researchers, and patient advocacy organizations can create a supportive environment that empowers patients to actively participate in their care decisions, access resources, and advocate for their needs. This comprehensive approach improves patient outcomes and contributes to reducing disparities in PCa.

## 6. Future Directions and Challenges

### 6.1. Multi-Disciplinary Collaborations

PCa continues to pose significant health disparities, necessitating a multi-disciplinary approach to improve outcomes and reduce disparities. Collaborations among various disciplines, including healthcare providers, researchers, public health professionals, community organizations, and policymakers, are vital for addressing PCa health disparities. Future directions and challenges in this area are numerous. Advancements in precision medicine hold promise for improving PCa outcomes. Collaborations between researchers, oncologists, and geneticists can further identify and validate biomarkers associated with PCa risk, prognosis, and response to treatment [230,231,232]. Such collaborations can facilitate the development of targeted therapies and personalized treatment approaches tailored to individual patients. Collaboration between healthcare systems and researchers is essential to implementing innovative strategies that improve access to high-quality care and reduce disparities [233,234]. This includes initiatives such as patient navigation programs, telehealth services, and community health worker interventions. By working together, healthcare systems and researchers can implement and evaluate these interventions to ensure their effectiveness in diverse populations. Multi-disciplinary collaborations involving community organizations, public health professionals, and researchers can foster community engagement and outreach initiatives. By understanding community needs, beliefs, and preferences, these collaborations can develop culturally appropriate interventions and educational campaigns. Such efforts can address barriers to early detection [235], screening, and treatment while also promoting health literacy and raising awareness about PCa disparities. Multi-disciplinary collaborations between researchers and policymakers are crucial for advocating policy changes that address PCa disparities. Collaboration can involve conducting and disseminating research findings, informing evidence-based policies, and advocating for increased funding for research, prevention, and treatment [236,237]. Engaging policymakers in dialogue can lead to the implementation of policies that support equitable access to care and reduce disparities. Collaboration between researchers, clinicians, and health systems is essential for sharing and integrating data to better understand PCa disparities. By pooling resources and data, researchers can conduct comprehensive analyses and identify patterns related to disparities [238,239]. Multi-disciplinary collaborations can also facilitate the development of standardized data collection methods and the establishment of registries to monitor and evaluate interventions targeting health disparities.

Challenges in multi-disciplinary collaborations include communication and coordination, resource allocation, institutional and cultural barriers, and sustainability. Effective collaboration requires clear communication and coordination among diverse stakeholders. Overcoming language barriers, varying expertise, and organizational structures can be challenging. Establishing effective communication channels and platforms for sharing information and fostering collaboration is crucial. Collaboration often necessitates the allocation of resources, including funding, staff, and infrastructure. Securing and distributing resources equitably among collaborating entities can be challenging. Ensuring adequate resources for research, intervention implementation, and sustainability is critical for successful multi-disciplinary collaborations. Different disciplines may have varying approaches, priorities, and institutional cultures. Overcoming disciplinary silos and bridging these gaps require mutual understanding, respect, and a willingness to work collaboratively. Building relationships and fostering a culture of collaboration among stakeholders can help address these barriers. Maintaining long-term collaborations and sustaining interventions beyond research projects can be challenging. Developing strategies for sustainability, including securing funding, training, and capacity-building, and establishing partnerships with community organizations, are essential for the long-term success of multi-disciplinary collaborations. In conclusion, multi-disciplinary collaborations are crucial for addressing PCa health disparities. Future directions involve precision medicine, health system innovations, community engagement, policy advocacy, and data integration. Overcoming challenges related to communication, resource allocation, institutional barriers, and sustainability is essential for successful collaborations. By working together, stakeholders can develop comprehensive strategies, implement effective interventions, and reduce PCa disparities, ultimately improving outcomes for all populations.

### 6.2. Precision Medicine and Personalized Care

Precision medicine, which involves tailoring medical treatments to individual patients based on their unique characteristics, holds significant potential for improving PCa outcomes and reducing health disparities. Future directions and challenges related to precision medicine and personalized care in addressing PCa health disparities remain in their infancy. Advancements in genomic profiling have identified specific genetic mutations and alterations that drive PCa [240,241]. Collaborations between researchers, oncologists, and geneticists can further refine our understanding of the genomic landscape of PCa, leading to the development of targeted therapies. Personalized treatment approaches based on specific genetic profiles can improve treatment outcomes and reduce disparities by ensuring that patients receive therapies that are most likely to be effective for their specific cancer subtype. Liquid biopsies, which involve the analysis of circulating tumor DNA or other biomarkers in bodily fluids, have shown promise for early detection and monitoring of PCa. Collaborations between researchers, clinicians, and diagnostic experts can advance the development and validation of liquid biopsy tests for early detection and screening [242,243]. Early detection is crucial for improving outcomes, as PCa is often diagnosed at advanced stages. Implementing personalized screening strategies based on individuals’ risk factors and genetic predispositions can help identify high-risk individuals and enable timely interventions. Collaborations between researchers and clinicians can identify predictive biomarkers that indicate the likelihood of response to specific treatments [244,245]. By understanding the genetic and molecular characteristics of pancreatic tumors, personalized treatment plans can be developed to optimize therapeutic responses. This approach can minimize unnecessary treatments, reduce toxicity, and improve outcomes for patients across different populations. Collaboration between researchers and bioinformatics experts can facilitate the integration of multi-omics data, such as genomics, transcriptomics, proteomics, and metabolomics, to gain a comprehensive understanding of PCa biology [246,247,248]. Integrative analysis of multi-omics data can provide insights into the underlying mechanisms, identify novel therapeutic targets, and guide personalized treatment strategies. Collaborations that combine expertise from diverse disciplines can harness the power of big data analytics and artificial intelligence to unlock new possibilities in personalized care. One of the key challenges in implementing precision medicine and personalized care is ensuring equitable access and affordability across diverse populations [249,250]. Collaborations involving policymakers, healthcare providers, patient advocates, and insurance companies are necessary to address these challenges. Efforts should focus on reducing barriers to access, ensuring coverage for genetic testing and targeted therapies, and promoting patient education and awareness about the benefits and availability of personalized care options. Precision medicine raises important ethical and privacy concerns, particularly regarding the collection, storage, and sharing of personal genomic data [251,252]. Collaborations among researchers, bioethicists, and legal experts are crucial for establishing robust frameworks and guidelines to address these concerns. Respecting patient autonomy, ensuring informed consent, and protecting patient privacy are essential to maintaining trust and promoting equitable access to personalized care. Integrating precision medicine approaches into routine clinical practice can be challenging. Collaboration between researchers, healthcare providers, and health systems is necessary to develop guidelines, establish clinical decision support tools, and provide necessary training and education to healthcare professionals. Additionally, collaborations can facilitate the translation of research findings into actionable clinical interventions, ensuring that personalized care becomes a standard of practice. In conclusion, precision medicine and personalized care have the potential to revolutionize PCa management and reduce health disparities. Future directions involve advancing genomic profiling, targeted therapies, liquid biopsies, and integrating multi-omics data. Overcoming challenges related to access, affordability, ethics, and implementation requires collaboration among stakeholders. By leveraging precision medicine approaches, we can enhance treatment outcomes, improve patient experiences, and ultimately address PCa health disparities.

### 6.3. Policy Changes and Health Equity

Policy changes and a focus on health equity are crucial for addressing PCa health disparities. Future directions involve advocating for increased research funding specifically allocated to PCa, with a focus on understanding the causes, risk factors, and mechanisms underlying the disease. Collaborations between researchers, patient advocacy groups, and policymakers can influence funding priorities and ensure that PCa research receives adequate support. Increased research funding can lead to advancements in early detection, treatment options, and targeted therapies. Policy changes should aim to improve access to and utilization of screening methods for PCa, particularly among high-risk populations. Collaboration between policymakers, healthcare providers, and professional societies can result in guidelines that promote regular screening for individuals with known risk factors, such as a family history of PCa or specific genetic mutations. Increasing awareness of early warning signs and symptoms can also aid in early detection efforts. Policy changes should focus on improving access to high-quality care for all individuals diagnosed with PCa. This includes ensuring that healthcare facilities and providers in underserved areas have the resources and expertise necessary to diagnose and treat PCa effectively. Cooperation between policymakers, healthcare systems, and community organizations can identify barriers to access and develop strategies to overcome them, such as expanding healthcare coverage, improving transportation options, and implementing telemedicine services. Policy changes should address health insurance disparities that contribute to unequal access to PCa care. Synergy between policymakers, insurers, and patient advocacy groups can promote reforms that provide comprehensive coverage for screening, diagnostic tests, treatment options, and supportive care services. Efforts should focus on reducing financial barriers, such as high out-of-pocket costs and pre-existing condition exclusions, to ensure equitable access to necessary care. Policy changes should emphasize the importance of culturally competent care in addressing PCa disparities. Partnerships between policymakers, healthcare providers, and community organizations can support initiatives that promote diversity and inclusion in the healthcare workforce, ensure language access to services, and address cultural barriers to care. Policies should encourage the development of educational programs that enhance healthcare providers’ cultural competence, enabling them to deliver personalized care that respects patients’ values, beliefs, and preferences. Policy changes should prioritize health literacy initiatives and patient education programs to improve understanding of PCa prevention, early detection, treatment options, and survivorship. Collaboration between policymakers, healthcare providers, and patient advocacy groups can develop and implement policies that promote clear and accessible health information, including educational materials, digital resources, and community-based workshops. Efforts should focus on reaching diverse populations, considering language, literacy levels, and cultural considerations. Addressing PCa disparities requires addressing the social determinants of health that contribute to unequal outcomes. Policy changes should target broader factors such as socioeconomic status, education, employment, and housing, which impact access to healthcare and influence health behaviors. Synergy between policymakers, public health experts, and community organizations can promote policies that address social determinants of health, ultimately leading to improved health equity.

Challenges in implementing policy changes and promoting health equity include overcoming political obstacles and garnering support for policy changes. This will require strong advocacy efforts. Collaboration between patient advocacy groups, researchers, healthcare providers, and policymakers is essential to driving policy changes that prioritize PCa disparities and health equity. Comprehensive data collection and surveillance systems are needed to monitor PCa disparities and evaluate the impact of policy changes. Collaboration between researchers, healthcare systems, and policymakers can facilitate the development of robust data infrastructure and standardized reporting mechanisms to track disparities over time and inform policy decisions. Addressing PCa disparities requires collaboration across sectors, including healthcare, public health, education, and social services. Collaboration between stakeholders from different sectors can be challenging due to differing priorities, structures, and funding streams. Efforts should be made to foster cross-sector collaborations, share resources, and align goals to promote health equity. Policy changes should be accompanied by rigorous evaluation to assess their effectiveness in reducing PCa disparities. Partnerships between researchers, policymakers, and healthcare providers are necessary to establish evaluation frameworks, measure outcomes, and hold stakeholders accountable for achieving health equity goals. In conclusion, policy changes and a focus on health equity are vital for addressing PCa health disparities. Future directions involve increasing research funding, promoting screening and early detection, enhancing access to quality care, addressing health insurance disparities, promoting culturally competent care, improving health literacy, and addressing social determinants of health. Overcoming challenges and fostering collaboration among stakeholders are essential for implementing effective policies and achieving health equity in PCa care.

### 6.4. Technological Innovations and Access

Technological innovations have the potential to play a significant role in addressing PCa health disparities by improving access to information, diagnosis, treatment, and supportive care. Future directions and challenges related to technological innovations and access in addressing PCa health disparities should be prioritized and tackled head-on. Telemedicine and virtual care technologies can enhance access to specialized healthcare services for individuals in underserved areas [253,254].

Collaboration between healthcare providers, technology developers, and policymakers can facilitate the implementation of telemedicine platforms that enable remote consultations, follow-up care, and multidisciplinary tumor boards [255]. However, challenges such as internet connectivity, access to digital devices, and privacy concerns need to be addressed to ensure equitable access to virtual care.

Collaborations between technology developers, healthcare providers, and researchers can lead to the development of mobile health applications (apps) that empower individuals to track symptoms, manage medications, access educational resources, and connect with support networks [256,257]. These apps can be particularly beneficial for individuals with limited access to healthcare facilities or those who face barriers to regular in-person visits. Ensuring the availability of user-friendly, culturally sensitive, and language-accessible apps is essential to addressing disparities in app usage and effectiveness.

Synergy between researchers, data scientists, and healthcare providers can harness the power of artificial intelligence (AI) and data analytics to improve PCa outcomes. AI algorithms can assist in early detection, risk prediction, treatment planning, and response monitoring [258,259]. However, challenges include the availability of diverse and representative datasets, addressing biases in AI algorithms, and integrating AI tools into clinical practice in an equitable and responsible manner.

Collaborations between healthcare systems, technology developers, and policymakers can promote the establishment of health information exchange networks [260]. These networks facilitate the secure and efficient exchange of patient data between healthcare providers, enabling seamless coordination of care and reducing the burden on patients to transfer medical records. Ensuring interoperability and data privacy while addressing concerns about data sharing are critical for equitable access to comprehensive patient information.

Wearable devices, such as smartwatches and fitness trackers, can enable continuous monitoring of vital signs, physical activity, and symptom progression. Collaboration between technology developers, researchers, and healthcare providers can leverage these devices to provide real-time data for early detection, personalized treatment monitoring, and patient self-management. Challenges include ensuring accuracy, reliability, and affordability of wearable devices, as well as addressing disparities in access to and utilization of these technologies [261,262].

Technological innovations can exacerbate health disparities if individuals lack digital literacy skills or face barriers to accessing technology. Collaboration between healthcare providers, community organizations, and policymakers is needed to bridge the digital divide by providing digital literacy training, ensuring affordability and availability of internet access, and tailoring technological solutions to diverse populations’ needs and preferences.

Policy changes and collaborations between policymakers, researchers, and technology developers are necessary to establish guidelines and regulations that ensure the ethical use of technology in PCa care. This includes addressing issues of privacy, security, informed consent, and transparency in data collection and utilization.

Addressing challenges related to technological innovations and access requires collaboration among stakeholders, including healthcare providers, technology developers, researchers, policymakers, and patient advocacy groups. Efforts should focus on promoting equity in technology development and implementation, addressing barriers to access, and ensuring that technological innovations are culturally sensitive, user-friendly, and effective across diverse populations. In conclusion, technological innovations have the potential to improve access and outcomes in PCa care. Future directions involve leveraging telemedicine, mobile health apps, AI, health information exchange, wearable devices, and remote monitoring [263]. However, challenges related to access, health literacy, data privacy, and regulatory considerations need to be addressed to ensure equitable and effective use of technology in addressing PCa health disparities.

## 7. Conclusions

In conclusion, this manuscript underscores the multifaceted nature of pancreatic cancer health disparities and the critical need for a comprehensive approach to address this pressing issue. The evidence and arguments presented throughout this work emphasize the intricate interplay of factors contributing to these health disparities, while pharmacologic anthropology provides a lens through which we can better understand and address them.

One of the pivotal facets of this issue lies in the genetic and biological factors influencing pancreatic cancer incidence and outcomes. Deepening our understanding of the genetic predispositions and molecular pathways associated with this disease is essential for developing targeted interventions and therapies that can reduce disparities in diagnosis, treatment outcomes, and even earlier detection.

Access to healthcare and treatment remains a central concern in addressing pancreatic cancer health disparities. Disparities in healthcare access, particularly in underserved communities, can significantly hinder early diagnosis and timely intervention. Efforts must be made to improve access to specialized care, ensure equitable resource allocation, and promote the availability of state-of-the-art treatments for all individuals affected by pancreatic cancer.

Socioeconomic factors play a pivotal role in perpetuating these disparities. Income inequality, education disparities, and economic instability can pose significant barriers to accessing healthcare services and adhering to treatment plans. Addressing the social determinants of health, such as employment opportunities and education, is crucial to reducing the burden of pancreatic cancer on marginalized populations and even the military.

Furthermore, enhanced patient-provider communications are essential to bridging the gap in healthcare disparities. Effective communication, rooted in cultural competence and empathy, fosters trust, shared decision-making, and treatment adherence. Healthcare providers must prioritize open and respectful dialogues with their patients, ensuring that information is accessible, comprehensible, and tailored to individual needs.

In light of these considerations, it is evident that addressing pancreatic cancer health disparities necessitates a multi-pronged approach. By integrating the insights of pharmacologic anthropology, we can develop strategies that account for the cultural, social, and biological factors at play. To strategically address these disparities, two key actions emerge:

1. Comprehensive Genetic and Biological Research: Invest in robust research initiatives that delve into the genetic and biological underpinnings of pancreatic cancer, aiming to identify risk factors, biomarkers, and therapeutic targets that can lead to more equitable treatment outcomes.
2. Equitable Access and Enhanced Communication: Advocate for policies and interventions that ensure equitable access to healthcare and treatment, while also emphasizing the importance of enhanced patient-provider communications, cultural competence, and shared decision-making in reducing disparities.

In conclusion, this manuscript underscores the urgency of addressing pancreatic cancer health disparities and offers a pathway forward through the integration of pharmacologic anthropology principles. By taking concrete steps to advance genetic and biological research, improve access to care, address socioeconomic determinants, and enhance patient-provider communications, we can move closer to the goal of achieving health equity for all individuals affected by pancreatic cancer.

## Figures and Tables

**Table 1:** Successful interventions in addressing pancreatic cancer health disparities.

Research Group	Action	Mission	Year	Citation
AACR	CBPR/CSI/PAE	To provide culturally tailored education, screening events, and support for early detection of PCa in African American Communities	1907	https://www.aacr.org/, accessed on 19 September 2023
Pancreatic Cancer Action Network (PanCAN)	CBPR/PAE	Advocacy to improve the lives of individuals with PCa while also raising awareness and funding for PCa.	1999	https://pancan.org/, accessed on 19 September 2023
the Patient Navigation in Medically Underserved Areas (PNMUA)	CSI/PAE	To provide improved access to treatment and promote early detection of cancer underserved populations, especially those with limited access to healthcare.	2005	[187,188]
Asian Liver Center at Stanford University	CBPR/CSI	To provide bilingual outreach and culturally appropriate education materials to the Asian community, to reduce the disparity of liver cancer.	1996	https://med.Stanford.edu/liver.html, accessed on 19 September 2023
High-Risk Pancreatic Cancer Clinic in theSkip Viragh Center for Pancreatic Cancer	CBPR/PAE	To initiate screenings on individuals with family history of PCa or known genetic mutations associated with PCa.	2003	https://www.hopkinsmedicine.org/kimmel-cancer-center/cancers-we-treat/pancreatic-cancer, accessed on 19 September 2023
The Detroit Urban Research Center (DURC)	CBPR	To conduct impactful research in the health disparities around Detroit and implement studies, community outreach programs, and policy advocacy efforts.	1995	[189,190]
The Appalachian Community Cancer Network (ACCN)	CBPR	To engage community members, healthcare providers, and researchers and identify community needs, develop interventions, and evaluate their effectiveness	2021	[194]
Latino Community Research (Various)	CBPR	To collaborate researchers, community organizations, health clinics, and individuals from Latino backgrounds to identify cultural beliefs, barriers to care, and information needs related to Pea.	NA	[191,192,193]
Project ECHO (Extension for Community Healthcare Outcomes)	CSI	To improve PCa care in underserved communities by connecting specialists with primary care providers and community health workers to deliver training, mentorship, and support in a culturally sensitive worldview.	2023	https://www.aap.org/en/practice-management/project-echo/, accessed on 19 September 2023
Faith Based Organizations (Various)	CSI	To promote partnerships with healthcare workers to facilitate community outreach, education, and support programs while also addressing the spiritual needs of the patrons.	NA	[202,203,204,205]
Community Health Workers (Various)	CSI/PAE	To mentor peers, patients and other community members to bridge the gap with cultural barriers and tailor innovations to meet specific cultural needs.	NA	[206,207,208]
Patient Support Groups/Online Communities and Forums	PAE	To inspire and empower patients by brining unique experiences and companionship to those currently suffering with the psychological and physical implications of cancer.	NA	[69,138]

CBPR = Community-Based Participatory Research; CSI = Culturally Sensitive Interventions; PAE = Patient Advocacy and Empowerment.

## References

[1] Afghani E., Klein A.P. (2022). Pancreatic Adenocarcinoma: Trends in Epidemiology, Risk Factors, and Outcomes. Hematol. Oncol. Clin. N. Am..

[2] Tavakkoli A., Singal A.G., Waljee A.K., Elmunzer B.J., Pruitt S.L., McKey T., Rubenstein J.H., Scheiman J.M., Murphy C.C. (2020). Racial Disparities and Trends in Pancreatic Cancer Incidence and Mortality in the United States. Clin. Gastroenterol. Hepatol..

[3] Chu Q.D., Hsieh M.C., Gibbs J.F., Wu X.C. (2021). Social determinants of health associated with poor outcome for rural patients following resected pancreatic cancer. J. Gastrointest. Oncol..

[4] DePalo D.K., Lee R.M., Lopez-Aguiar A.G., Gamboa A.C., Rocha F., Poultsides G., Dillhoff M., Fields R.C., Idrees K., Nathan H. (2019). Interaction of race and pathology for neuroendocrine tumors: Epidemiology, natural history, or racial disparity?. J. Surg. Oncol..

[5] Gad M.M., Saad A.M., Al-Husseini M.J., Abdel-Gawad Y.M., Alsalhani O.M., Alhaddad R., Mohamad B., Saleh M.A., Simons-Linares C.R. (2020). Temporal trends of pancreatic ductal adenocarcinoma in young adults in the United States: A Population-Based Study. Clin. Res. Hepatol. Gastroenterol..

[6] Scarton L., Yoon S., Oh S., Agyare E., Trevino J., Han B., Lee E., Setiawan V.W., Permuth J.B., Schmittgen T.D. (2018). Pancreatic Cancer Related Health Disparities: A Commentary. Cancers.

[7] Ahuja A., Yi Y.S., Kim M.Y., Cho J.Y. (2018). Ethnopharmacological properties of Artemisia asiatica: A comprehensive review. J. Ethnopharmacol..

[8] Liu X., Li Z., Wang Y. (2021). Advances in Targeted Therapy and Immunotherapy for Pancreatic Cancer. Adv. Biol..

[9] Sun J., Russell C.C., Scarlett C.J., McCluskey A. (2020). Small molecule inhibitors in pancreatic cancer. RSC Med. Chem..

[10] Martin A.N., Snyder R.A. (2023). Racial disparities in pancreatic cancer clinical trials: Defining the problem and identifying solutions. Adv. Cancer Res..

[11] Katona B.W., Klute K., Brand R.E., Everett J.N., Farrell J.J., Hawthorne K., Kaul V., Kupfer S.S., Paiella S., Simeone D.M. (2023). Racial, Ethnic, and Sex-based Disparities among High-risk Individuals Undergoing Pancreatic Cancer Surveillance. Cancer Prev. Res..

[12] Brooks G.A., Tomaino M.R., Ramkumar N., Wang Q., Kapadia N.S., O’Malley A.J., Wong S.L., Loehrer A.P., Tosteson A.N.A. (2023). Association of rurality, socioeconomic status, and race with pancreatic cancer surgical treatment and survival. J. Natl. Cancer Inst..

[13] Halder R., Veeravelli S., Cheng C., Estrada-Mendizabal R.J., Recio-Boiles A. (2023). Health Disparities in Presentation, Treatment, Genomic Testing, and Outcomes of Pancreatic Cancer in Hispanic and Non-Hispanic Patients. J. Racial Ethn. Health Disparities.

[14] Behar-Horenstein L., Warren R.C., Setiawan V.W., Perkins C., Schmittgen T.D. (2020). Enhancing African American Participation in Biospecimens: A Case in Point for Pancreatic Cancer. Cancer Health Disparities.

[15] Xu C., Wang Y., Yang H., Hou J., Sun L., Zhang X., Cao X., Hou Y., Wang L., Cai Q. (2019). Association Between Cancer Incidence and Mortality in Web-Based Data in China: Infodemiology Study. J. Med. Internet Res..

[16] Coffin T.B., Kenner B.J. (2022). Challenges in Recruitment and Retention: Leveraging Health-Related Antecedents and Information Carrier Factors to Improve Patient Participation in Pancreatic Cancer Research-A Review Article. Pancreas.

[17] Visser B.C., Ma Y., Zak Y., Poultsides G.A., Norton J.A., Rhoads K.F. (2012). Failure to comply with NCCN guidelines for the management of pancreatic cancer compromises outcomes. HPB.

[18] Lin Y.J., Burkhart R., Lu T.P., Wolfgang C., Wright M., Zheng L., Wu H.Y., Chen C.H., Lee S.Y., Wu C.H. (2022). Solid Pseudopapillary Neoplasms of the Pancreas Across Races Demonstrate Disparities with Comparably Good Prognosis. World J. Surg..

[19] Silverman D.T., Hoover R.N., Brown L.M., Swanson G.M., Schiffman M., Greenberg R.S., Hayes R.B., Lillemoe K.D., Schoenberg J.B., Schwartz A.G. (2003). Why do Black Americans have a higher risk of pancreatic cancer than White Americans?. Epidemiology.

[20] Fabregas J.C., Riley K.E., Brant J.M., George T.J., Orav E.J., Lam M.B. (2022). Association of social determinants of health with late diagnosis and survival of patients with pancreatic cancer. J. Gastrointest. Oncol..

[21] Hao S., Mitsakos A., Irish W., Tuttle-Newhall J.E., Parikh A.A., Snyder R.A. (2022). Differences in receipt of multimodality therapy by race, insurance status, and socioeconomic disadvantage in patients with resected pancreatic cancer. J. Surg. Oncol..

[22] Mehta V.V., Friedmann P., McAuliffe J.C., Muscarella P., In H. (2021). Pancreatic Cancer Surgery Following Emergency Department Admission: Understanding Poor Outcomes and Disparities in Care. J. Gastrointest. Surg..

[23] Riner A.N., Underwood P.W., Yang K., Herremans K.M., Cameron M.E., Chamala S., Qiu P., George T.J., Permuth J.B., Merchant N.B. (2020). Disparities in Pancreatic Ductal Adenocarcinoma-The Significance of Hispanic Ethnicity, Subgroup Analysis, and Treatment Facility on Clinical Outcomes. Cancer Med..

[24] Moaven O., Richman J.S., Reddy S., Wang T., Heslin M.J., Contreras C.M. (2019). Healthcare disparities in outcomes of patients with resectable pancreatic cancer. Am. J. Surg..

[25] Seyedin S., Luu C., Stabile B.E., Lee B. (2012). Effect of socioeconomic status on surgery for pancreatic adenocarcinoma. Am. Surg..

[26] Chang C.M., Su Y.C., Lai N.S., Huang K.Y., Chien S.H., Chang Y.H., Lian W.C., Hsu T.W., Lee C.C. (2012). The combined effect of individual and neighborhood socioeconomic status on cancer survival rates. PLoS ONE.

[27] Powell C.B., Laurent C., Garcia C., Hoodfar E., Karlea A., Kobelka C., Lee J., Roh J., Kushi L.H. (2022). Factors influencing genetic counseling and testing for hereditary breast and ovarian cancer syndrome in a large US health care system. Clin. Genet..

[28] Zavala V.A., Bracci P.M., Carethers J.M., Carvajal-Carmona L., Coggins N.B., Cruz-Correa M.R., Davis M., de Smith A.J., Dutil J., Figueiredo J.C. (2021). Cancer health disparities in racial/ethnic minorities in the United States. Br. J. Cancer.

[29] Ashktorab H., Kupfer S.S., Brim H., Carethers J.M. (2017). Racial Disparity in Gastrointestinal Cancer Risk. Gastroenterology.

[30] Liu C.T., Raghavan S., Maruthur N., Kabagambe E.K., Hong J., Ng M.C., Hivert M.F., Lu Y., An P., Bentley A.R. (2016). Trans-ethnic Meta-analysis and Functional Annotation Illuminates the Genetic Architecture of Fasting Glucose and Insulin. Am. J. Hum. Genet..

[31] Singal V., Singal A.K., Kuo Y.F. (2012). Racial disparities in treatment for pancreatic cancer and impact on survival: A population-based analysis. J. Cancer Res. Clin. Oncol..

[32] Gordon D.R., Radecki Breitkopf C., Robinson M., Petersen W.O., Egginton J.S., Chaffee K.G., Petersen G.M., Wolf S.M., Koenig B.A. (2019). Should Researchers Offer Results to Family Members of Cancer Biobank Participants? A Mixed-Methods Study of Proband and Family Preferences. AJOB Empir. Bioeth..

[33] Jackson F.L. (2006). Illuminating cancer health disparities using ethnogenetic layering (EL) and phenotype segregation network analysis (PSNA). J. Cancer Educ..

[34] Mitsakos A.T., Dennis S.O., Parikh A.A., Snyder R.A. (2021). Thirty-day complication rates do not differ by race among patients undergoing pancreaticoduodenectomy for pancreatic adenocarcinoma. J. Surg. Oncol..

[35] Salami A., Alvarez N.H., Joshi A.R.T. (2017). Geographic disparities in surgical treatment recommendation patterns and survival for pancreatic adenocarcinoma. HPB.

[36] Abbott D.E., Martin G., Kooby D.A., Merchant N.B., Squires M.H., Maithel S.K., Weber S.M., Winslow E.R., Cho C.S., Bentrem D.J. (2016). Perception Is Reality: Quality metrics in pancreas surgery—A Central Pancreas Consortium (CPC) analysis of 1399 patients. HPB.

[37] Diaz Osterman C.J., Gonda A., Stiff T., Sigaran U., Valenzuela M.M., Ferguson Bennit H.R., Moyron R.B., Khan S., Wall N.R. (2016). Curcumin Induces Pancreatic Adenocarcinoma Cell Death Via Reduction of the Inhibitors of Apoptosis. Pancreas.

[38] Galloway N.R., Aspe J.R., Sellers C., Wall N.R. (2009). Enhanced antitumor effect of combined gemcitabine and proton radiation in the treatment of pancreatic cancer. Pancreas.

[39] Gleason-Comstock J., Calhoun C.B., Mozeb G., Louis C., Hill A., Locke B.J., Harrell V., Yasmin S., Zhang L., Flack J.M. (2023). Recruitment, Retention, and Future Direction for a Heart Health Education and Risk Reduction Intervention Led by Community Health Workers in an African American Majority City. J. Racial Ethn. Health Disparities.

[40] Joo W.T. (2023). Educational gradient in social network changes at disease diagnosis. Soc. Sci. Med..

[41] Hall J.M., Chakrabarti C., Mkuu R., Thompson L.A., Shenkman E.A., Theis R.P. (2023). The Association of Socioeconomic Vulnerability and Race and Ethnicity with Disease Burden among Children in a Statewide Medicaid Population. Acad. Pediatr..

[42] Jones B.L., Carter M.C., Davis C.M., Wang J. (2023). Diversity, Equity, and Inclusion: A Decade of Progress?. J. Allergy Clin. Immunol. Pract..

[43] Rafizadeh E.B., Rice E., Smith J., Bell J., Harvath T.A. (2023). Understanding How Community Health Workers Build Trust with Low-Income Women of Color At-Risk for Maternal Child Health Disparities: A Grounded Theory Study. J. Community Health Nurs..

[44] Kim G., Qin J., Hall C.B., In H. (2022). Association Between Socioeconomic and Insurance Status and Delayed Diagnosis of Gastrointestinal Cancers. J. Surg. Res..

[45] Zhu F., Wang H., Ashamalla H. (2020). Racial and Socioeconomic Disparities in the Treatments and Outcomes of Pancreatic Cancer among Different Treatment Facility Types. Pancreas.

[46] Noel M., Fiscella K. (2019). Disparities in Pancreatic Cancer Treatment and Outcomes. Health Equity.

[47] Vallance A.E., van der Meulen J., Kuryba A., Braun M., Jayne D.G., Hill J., Cameron I.C., Walker K. (2018). Socioeconomic differences in selection for liver resection in metastatic colorectal cancer and the impact on survival. Eur. J. Surg. Oncol..

[48] Shapiro M., Chen Q., Huang Q., Boosalis V.A., Yoon C.H., Saund M.S., Whang E.E., Gold J.S. (2016). Associations of Socioeconomic Variables with Resection, Stage, and Survival in Patients with Early-Stage Pancreatic Cancer. JAMA Surg..

[49] Bakens M., Lemmens V., de Hingh I. (2017). Socio-economic status influences the likelihood of undergoing surgical treatment for pancreatic cancer in the Netherlands. HPB.

[50] Del Valle J.P., Fillmore N.R., Molina G., Fairweather M., Wang J., Clancy T.E., Ashley S.W., Urman R.D., Whang E.E., Gold J.S. (2022). Socioeconomic Disparities in Pancreas Cancer Resection and Survival in the Veterans Health Administration. Ann. Surg. Oncol..

[51] Gallegos J.M., Taylor A., Vardell V., Silberstein P.T. (2023). Socioeconomic Factors Associated with a Late-Stage Pancreatic Cancer Diagnosis: An Analysis of the National Cancer Database. Cureus.

[52] Torres M.B., Dixon M.E.B., Gusani N.J. (2022). Undertreatment of Pancreatic Cancer: The Intersection of Bias, Biology, and Geography. Surg. Oncol. Clin. N. Am..

[53] Underwood P.W., Riner A.N., Neal D., Cameron M.E., Yakovenko A., Reddy S., Rose J.B., Hughes S.J., Trevino J.G. (2021). It’s more than just cancer biology: Health disparities in patients with pancreatic neuroendocrine tumors. J. Surg. Oncol..

[54] Liu J.H., Zingmond D.S., McGory M.L., SooHoo N.F., Ettner S.L., Brook R.H., Ko C.Y. (2006). Disparities in the utilization of high-volume hospitals for complex surgery. JAMA.

[55] Boonhat H., Pratama A.P., Lin J.T., Lin R.T. (2023). Duration-response association between occupational exposure and pancreatic cancer risk: Meta-analysis. Occup. Med..

[56] Kamendulis L.M., Hocevar J.M., Stephens M., Sandusky G.E., Hocevar B.A. (2022). Exposure to perfluorooctanoic acid leads to promotion of pancreatic cancer. Carcinogenesis.

[57] Togawa K., Leon M.E., Lebailly P., Beane Freeman L.E., Nordby K.C., Baldi I., MacFarlane E., Shin A., Park S., Greenlee R.T. (2021). Cancer incidence in agricultural workers: Findings from an international consortium of agricultural cohort studies (AGRICOH). Environ. Int..

[58] Endo M., Haruyama Y., Muto G., Kiyohara K., Mizoue T., Kojimahara N., Yamaguchi N. (2018). Work Sustainability among Male Cancer Survivors after Returning to Work. J. Epidemiol..

[59] de Boer A.G., Bruinvels D.J., Tytgat K.M., Schoorlemmer A., Klinkenbijl J.H., Frings-Dresen M.H. (2011). Employment status and work-related problems of gastrointestinal cancer patients at diagnosis: A cross-sectional study. BMJ Open.

[60] Zaitsu M., Kim Y., Lee H.E., Takeuchi T., Kobayashi Y., Kawachi I. (2019). Occupational class differences in pancreatic cancer survival: A population-based cancer registry-based study in Japan. Cancer Med..

[61] Munigala S., Gardner T.B., O’Reilly E.M., Fernandez-Del Castillo C., Ko A.H., Pleskow D., Vollmer C.M., Searle N.A., Bakelman D., Holt J.M. (2022). Helping Patients Understand Pancreatic Cancer Using Animated Pancreas Patient Education with Visual Formats of Learning. Pancreas.

[62] Humphrys E., Burt J., Rubin G., Emery J.D., Walter F.M. (2019). The influence of health literacy on the timely diagnosis of symptomatic cancer: A systematic review. Eur. J. Cancer Care.

[63] Munigala S., Gardner T.B., O’Reilly E.M., Castillo C.F., Ko A.H., Pleskow D., Mills J.B., Vollmer C.M., Searle N.A., Alsante M. (2018). Understanding Pancreatic Diseases Using Animated Pancreas Patient: Informing Patients for Better Health Outcomes with Visual Formats of Learning. Pancreas.

[64] Garland M.E., Lukac D., Contreras P. (2023). A Brief Report: Comparative Evaluation of Online Spanish and English Content on Pancreatic Cancer Treatment. J. Cancer Educ..

[65] Riner A.N., Girma S., Vudatha V., Mukhopadhyay N., Skoro N., Gal T.S., Freudenberger D.C., Herremans K.M., George T.J., Trevino J.G. (2022). Eligibility Criteria Perpetuate Disparities in Enrollment and Participation of Black Patients in Pancreatic Cancer Clinical Trials. J. Clin. Oncol..

[66] Paltoo D.N., Chu K.C. (2004). Patterns in cancer incidence among American Indians/Alaska Natives, United States, 1992–1999. Public Health Rep..

[67] Francis D.B., Zelaya C.M. (2021). Cancer Fatalism and Cancer Information Seeking among Black Women: Examining the Impact of Aretha Franklin’s Death on Cancer Communication Outcomes. J. Cancer Educ..

[68] Rauscher E.A., Dean M., Campbell-Salome G.M. (2018). “I Am Uncertain about What My Uncertainty Even Is”: Men’s Uncertainty and Information Management of Their BRCA-Related Cancer Risks. J. Genet. Couns..

[69] Foroughi F., Lam A.K., Lim M.S.C., Saremi N., Ahmadvand A. (2016). “Googling” for Cancer: An Infodemiological Assessment of Online Search Interests in Australia, Canada, New Zealand, the United Kingdom, and the United States. JMIR Cancer.

[70] Kim N., Kang D., Shin S.H., Heo J.S., Shim S., Lim J., Cho J., Han I.W. (2023). Effects of cancer stigma on quality of life of patients with hepatobiliary and pancreatic cancer. Ann. Hepatobiliary Pancreat Surg..

[71] Warner E.T., Park E.R., Luberto C.M., Rabin J., Perez G.K., Ostroff J.S. (2022). Internalized stigma among cancer patients enrolled in a smoking cessation trial: The role of cancer type and associations with psychological distress. Psychooncology.

[72] Cabasag C.J., Arnold M., Pineros M., Morgan E., Brierley J., Hofferkamp J., Kehoe S., Butler J., Bucher O., Bray F. (2021). Population-based cancer staging for oesophageal, gastric, and pancreatic cancer 2012–2014: International Cancer Benchmarking Partnership SurvMark-2. Int. J. Cancer.

[73] Tang D., Tang Q., Huang W., Zhang Y., Tian Y., Fu X. (2023). Fasting: From Physiology to Pathology. Adv. Sci..

[74] Jiang Y., Sohal D.P.S. (2023). Pancreatic Adenocarcinoma Management. JCO Oncol. Pract..

[75] Chung V., Sun V., Ruel N., Smith T.J., Ferrell B.R. (2022). Improving Palliative Care and Quality of Life in Pancreatic Cancer Patients. J. Palliat. Med..

[76] Park S., Ortega A.N., Chen J., Bustamante A.V. (2023). Effects of Medicare eligibility and enrollment at age 65 among immigrants and US-born residents. J. Am. Geriatr. Soc..

[77] Malagon T., Morais S., Tope P., El-Zein M., Franco E.L. (2023). Site-specific cancer incidence by race and immigration status in Canada 2006-2015: A population-based data linkage study. Cancer Epidemiol. Biomark. Prev..

[78] La Frinere-Sandoval Q., Cubbin C., DiNitto D.M. (2023). Racial and ethnic disparities in cervical and breast cancer screenings by nativity and length of U.S. residence. Ethn. Health.

[79] Murrar S., Baqai B., Padela A.I. (2023). Predictors of Perceived Discrimination in Medical Settings among Muslim Women in the USA. J. Racial Ethn. Health Disparities.

[80] Zhang M., Sit J.W.H., Choi K.C., Chan C.W.H. (2023). A theory-driven, culture-tailored educational intervention for cervical cancer screening among rural Chinese females: A feasibility and pilot study. Asia Pac. J. Oncol. Nurs..

[81] Mishra S.I., Adsul P., Leekity S., Rodman J., Sussman A.L., Kelly K., Sheche J., Faber T., Shah V. (2023). A culturally informed model to enhance breast, cervical, and colorectal cancer screenings: Perspectives of American Indian adults and healthcare providers in rural New Mexico. Cancer Causes Control.

[82] Roh S., Lee Y.S. (2023). Developing Culturally Tailored Mobile Web App Education to Promote Breast Cancer Screening: Knowledge, Barriers, and Needs among American Indian Women. J. Cancer Educ..

[83] Richardson-Parry A., Silva M., Valderas J.M., Donde S., Woodruff S., van Vugt J. (2023). Interactive or tailored digital interventions to increase uptake in cervical, breast, and colorectal cancer screening to reduce health inequity: A systematic review. Eur. J. Cancer Prev..

[84] Cobianchi L., Dal Mas F., Denicolai S., Previtali P., Venturi A. (2023). Editorial: New frontiers in pancreatic cancer care: Multidisciplinary approaches, the role of Pancreas Units, and their organizational impacts. Front. Surg..

[85] Kolbeinsson H.M., Chandana S., Wright G.P., Chung M. (2023). Pancreatic Cancer: A Review of Current Treatment and Novel Therapies. J. Investig. Surg..

[86] Khan N.N., Evans S.M., Ioannou L.J., Pilgrim C.H.C., Blanchard M., Daveson B., Philip J., Zalcberg J.R., Te Marvelde L. (2023). Characteristics of patients diagnosed with pancreatic cancer who access palliative care: An observational study. Qual. Life Res..

[87] Soreide K., Ismail W., Roalso M., Ghotbi J., Zaharia C. (2023). Early Diagnosis of Pancreatic Cancer: Clinical Premonitions, Timely Precursor Detection and Increased Curative-Intent Surgery. Cancer Control.

[88] Digiacomo L., Quagliarini E., Pozzi D., Coppola R., Caracciolo G., Caputo D. (2023). Stratifying Risk for Pancreatic Cancer by Multiplexed Blood Test. Cancers.

[89] He Y., Huang W., Tang Y., Li Y., Peng X., Li J., Wu J., You N., Li L., Liu C. (2023). Clinical and genetic characteristics in pancreatic cancer from Chinese patients revealed by whole exome sequencing. Front. Oncol..

[90] Huang Y., Zhou S., Luo Y., Zou J., Li Y., Chen S., Gao M., Huang K., Lian G. (2023). Development and validation of a radiomics model of magnetic resonance for predicting liver metastasis in resectable pancreatic ductal adenocarcinoma patients. Radiat. Oncol..

[91] Li J., Huang L., Liao C., Liu G., Tian Y., Chen S. (2023). Two machine learning-based nomogram to predict risk and prognostic factors for liver metastasis from pancreatic neuroendocrine tumors: A multicenter study. BMC Cancer.

[92] Lippman S.M., Abate-Shen C., Colbert Maresso K.L., Colditz G.A., Dannenberg A.J., Davidson N.E., Disis M.L., DuBois R.N., Szabo E., Giuliano A.R. (2018). AACR White Paper: Shaping the Future of Cancer Prevention—A Roadmap for Advancing Science and Public Health. Cancer Prev. Res..

[93] Han X., Zhao J., Yabroff K.R., Johnson C.J., Jemal A. (2022). Association Between Medicaid Expansion under the Affordable Care Act and Survival among Newly Diagnosed Cancer Patients. J. Natl. Cancer Inst..

[94] Mellado S., Vega E.A., Abudalou M., Kutlu O.C., Salehi O., Li M., Kozyreva O., Freeman R., Conrad C. (2022). Trends in Preoperative Chemotherapy Utilization for Proximal Pancreatic Cancer: Are We Making Progress?. J. Gastrointest. Surg..

[95] Ueberroth B.E., Khan A., Zhang K.J., Philip P.A. (2021). Differences in Baseline Characteristics and White Blood Cell Ratios Between Racial Groups in Patients with Pancreatic Adenocarcinoma. J. Gastrointest. Cancer.

[96] Robbins H.A., Engels E.A., Pfeiffer R.M., Shiels M.S. (2015). Age at cancer diagnosis for blacks compared with whites in the United States. J. Natl. Cancer Inst..

[97] Macklin-Mantia S.K., Clift K.E., Maimone S., Hodge D.O., Riegert-Johnson D., Hines S.L. (2023). Patient uptake of updated genetic testing following uninformative BRCA1 and BRCA2 results. J. Genet. Couns..

[98] Zhu H., Welinsky S., Soper E.R., Brown K.L., Abul-Husn N.S., Lucas A.L. (2021). Genetic Variants in Patients with a Family History of Pancreatic Cancer: Impact of Multigene Panel Testing. Pancreas.

[99] Abe K., Ueki A., Urakawa Y., Kitago M., Yoshihama T., Nanki Y., Kitagawa Y., Aoki D., Kosaki K., Hirasawa A. (2021). Familial pancreatic cancer with PALB2 and NBN pathogenic variants: A case report. Hered. Cancer Clin. Pract..

[100] Nakamura K., Zhu Z., Roy S., Jun E., Han H., Munoz R.M., Nishiwada S., Sharma G., Cridebring D., Zenhausern F. (2022). An Exosome-based Transcriptomic Signature for Noninvasive, Early Detection of Patients with Pancreatic Ductal Adenocarcinoma: A Multicenter Cohort Study. Gastroenterology.

[101] Chung C., Galvin R., Achenbach E., Dziadkowiec O., Sen S. (2021). Characterization of Blood-Based Molecular Profiling in Pancreatic Adenocarcinoma. Oncology.

[102] Donahue T.R., Tran L.M., Hill R., Li Y., Kovochich A., Calvopina J.H., Patel S.G., Wu N., Hindoyan A., Farrell J.J. (2012). Integrative survival-based molecular profiling of human pancreatic cancer. Clin. Cancer Res..

[103] Zou Y., Pitchumoni C.S. (2023). Obesity, obesities and gastrointestinal cancers. Dis. Mon..

[104] Neumann C.C.M., Schneider F., Hilfenhaus G., Vecchione L., Benzing C., Ihlow J., Fehrenbach U., Malinka T., Keilholz U., Stintzing S. (2023). Impact of Smoking, Body Weight, Diabetes, Hypertension and Kidney Dysfunction on Survival in Pancreatic Cancer Patients-A Single Center Analysis of 2323 Patients within the Last Decade. J. Clin. Med..

[105] Nodari Y., Gentiluomo M., Mohelnikova-Duchonova B., Kreivenaite E., Milanetto A.C., Skieceviciene J., Landi S., Lawlor R.T., Petrone M.C., Arcidiacono P.G. (2023). Genetic and non-genetic risk factors for early-onset pancreatic cancer. Dig. Liver Dis..

[106] Perez-Diez I., Andreu Z., Hidalgo M.R., Perpina-Clerigues C., Fantin L., Fernandez-Serra A., de la Iglesia-Vaya M., Lopez-Guerrero J.A., Garcia-Garcia F. (2023). A Comprehensive Transcriptional Signature in Pancreatic Ductal Adenocarcinoma Reveals New Insights into the Immune and Desmoplastic Microenvironments. Cancers.

[107] Osman K., Ahmet K., Hilmi T., Ilker N.O., Ercan O., Devrim C., Murat S., Emre C., Ilhan H., Mustafa G. (2023). BRCA 1/BRCA 2 Pathogenic/Likely Pathogenic Variant Patients with Breast, Ovarian, and Other Cancers. Balk. J. Med. Genet..

[108] Clark N.M., Roberts E.A., Fedorenko C., Sun Q., Dubard-Gault M., Handford C., Yung R., Cheng H.H., Sham J.G., Norquist B.M. (2023). Genetic Testing among Patients with High-Risk Breast, Ovarian, Pancreatic, and Prostate Cancers. Ann. Surg. Oncol..

[109] Everett J.N., Dettwyler S.A., Jing X., Stender C., Schmitter M., Baptiste A., Chun J., Kawaler E.A., Khanna L.G., Gross S.A. (2023). Impact of comprehensive family history and genetic analysis in the multidisciplinary pancreatic tumor clinic setting. Cancer Med..

[110] Dayyani F., Macarulla T., Johnson A., Wainberg Z.A. (2023). Second-line treatment options for patients with metastatic pancreatic ductal adenocarcinoma: A systematic literature review. Cancer Treat. Rev..

[111] Kitamura H., Nakazawa J., Nagashima F., Andou M., Furuse J. (2023). The Prognostic Utility of a Geriatric Assessment for Patients with Pancreatic Cancer Receiving Gemcitabine-based Chemotherapy: A Prospective Observational Study. Intern. Med..

[112] Farrell J.J., Robert M.E., Lacy J. (2023). Precision Medicine for Pancreas Cancer Treatment: A Multidisciplinary Challenge or Opportunity?. Clin. Gastroenterol. Hepatol..

[113] Molnar A., Monroe H., Basri Aydin H., Arslan M.E., Lightle A., Lee H., El Jabbour T. (2023). Tumors of the Digestive System: Comprehensive Review of Ancillary Testing and Biomarkers in the Era of Precision Medicine. Curr. Oncol..

[114] Sereti E., Papapostolou I., Dimas K. (2023). Pancreatic Cancer Organoids: An Emerging Platform for Precision Medicine?. Biomedicines.

[115] Belleau P., Deschenes A., Chambwe N., Tuveson D.A., Krasnitz A. (2023). Genetic Ancestry Inference from Cancer-Derived Molecular Data across Genomic and Transcriptomic Platforms. Cancer Res..

[116] Hamada T., Yuan C., Yurgelun M.B., Perez K., Khalaf N., Morales-Oyarvide V., Babic A., Nowak J.A., Rubinson D.A., Giannakis M. (2019). Family history of cancer, Ashkenazi Jewish ancestry, and pancreatic cancer risk. Br. J. Cancer.

[117] Adams A.M., Reames B.N., Krell R.W. (2023). Morbidity and Mortality of Non-pancreatectomy operations for pancreatic cancer: An ACS-NSQIP analysis. Am. J. Surg..

[118] Fraumeni J.F. (1975). Cancers of the pancreas and biliary tract: Epidemiological considerations. Cancer Res..

[119] Del Nero L., Dabizzi E., Ceglie A., Ziola S., Zerbi A., Baron T.H., Conio M. (2023). Familial pancreatic cancer. Clin. Res. Hepatol. Gastroenterol..

[120] Amaral M.J., Oliveira R.C., Donato P., Tralhao J.G. (2023). Pancreatic Cancer Biomarkers: Oncogenic Mutations, Tissue and Liquid Biopsies, and Radiomics-A Review. Dig. Dis. Sci..

[121] Balzano G., Guarneri G., Pecorelli N., Partelli S., Crippa S., Vico A., Falconi M., Baglio G. (2023). Geographical Disparities and Patients’ Mobility: A Plea for Regionalization of Pancreatic Surgery in Italy. Cancers.

[122] Calvillo-Ortiz R., Polanco-Santana J.C., Castillo-Angeles M., Allar B.G., Anguiano-Landa L., Ghaffarpasand E., Barrows C., Callery M.P., Kent T.S. (2022). Language Proficiency and Survival in Pancreatic Cancer: A Propensity Score-Matched Analysis. J. Gastrointest. Surg..

[123] Mobley E.M., Guerrier C., Tfirn I., Gutter M.S., Vigal K., Pather K., Braithwaite D., Nataliansyah M.M., Tsai S., Baskovich B. (2023). Impact of Medicaid Expansion on Stage at Diagnosis for US Adults with Pancreatic Cancer: A Population-Based Study. J. Racial Ethn. Health Disparities.

[124] Fong Z.V., Teinor J., Yeo T.P., Rinaldi D., Greer J.B., Lavu H., Qadan M., Johnston F.M., Ferrone C.R., Chang D.C. (2023). Profile of the Postoperative Care Provided for Patients with Pancreatic and Periampullary Cancers by Family and Unpaid Caregivers. JCO Oncol. Pract..

[125] Permuth J.B., Clark Daly A., Jeong D., Choi J.W., Cameron M.E., Chen D.T., Teer J.K., Barnett T.E., Li J., Powers B.D. (2019). Racial and ethnic disparities in a state-wide registry of patients with pancreatic cancer and an exploratory investigation of cancer cachexia as a contributor to observed inequities. Cancer Med..

[126] Bogumil D., Conti D.V., Sheng X., Xia L., Shu X.O., Pandol S.J., Blot W.J., Zheng W., Le Marchand L., Haiman C.A. (2020). Replication and Genetic Risk Score Analysis for Pancreatic Cancer in a Diverse Multiethnic Population. Cancer Epidemiol. Biomark. Prev..

[127] Hiltemann S., Rasche H., Gladman S., Hotz H.R., Lariviere D., Blankenberg D., Jagtap P.D., Wollmann T., Bretaudeau A., Goue N. (2023). Galaxy Training: A powerful framework for teaching!. PLoS Comput. Biol..

[128] van der Horst D.E.M., Garvelink M.M., Bos W.J.W., Stiggelbout A.M., Pieterse A.H. (2023). For which decisions is Shared Decision Making considered appropriate?—A systematic review.. Patient Educ. Couns..

[129] Freund K.M., Reisinger S.A., LeClair A.M., Yoon G.H., Al-Najar S.M., Young G.S., Gonzalez E.T., Oliveri J.M., Paskett E.D. (2019). Insurance Stability and Cancer Screening Behaviors. Health Equity.

[130] Karagoz M.A., Sarica K. (2023). Patient compliance to dietary recommendations: Tips and tricks to improve compliance rates. World J. Urol..

[131] Shahid S., Durey A., Bessarab D., Aoun S.M., Thompson S.C. (2013). Identifying barriers and improving communication between cancer service providers and Aboriginal patients and their families: The perspective of service providers. BMC Health Serv. Res..

[132] Landers A., McKenzie C., Pitama S.G., Brown H. (2020). Enzyme replacement in advanced pancreatic cancer: Patient perceptions. BMJ Support. Palliat. Care.

[133] Andersson T.K., Engstrom M., Bjersa K. (2022). Perceptions of Experiences of Recovery after Pancreaticoduodenectomy-A Phenomenographic Interview Study. Cancer Nurs..

[134] Schildmann J., Ritter P., Salloch S., Uhl W., Vollmann J. (2013). ‘One also needs a bit of trust in the doctor... ’: A qualitative interview study with pancreatic cancer patients about their perceptions and views on information and treatment decision-making. Ann. Oncol..

[135] Weisburger J.H. (2000). Approaches for chronic disease prevention based on current understanding of underlying mechanisms. Am. J. Clin. Nutr..

[136] Eigenschink M., Bellach L., Leonard S., Dablander T.E., Maier J., Dablander F., Sitte H.H. (2023). Cross-sectional survey and Bayesian network model analysis of traditional Chinese medicine in Austria: Investigating public awareness, usage determinants and perception of scientific support. BMJ Open.

[137] Ikemoto T., Sugimoto K., Takita M., Shimoda M., Noguchi H., Naziruddin B., Levy M.F., Shimada M., Matsumoto S. (2011). Japanese herbal medicine TJ-48 prevents autoimmune diabetes in NOD mice. Am. J. Chin. Med..

[138] Li L., Leung P.S. (2014). Use of herbal medicines and natural products: An alternative approach to overcoming the apoptotic resistance of pancreatic cancer. Int. J. Biochem. Cell Biol..

[139] Nie J., Zhao C., Deng L.I., Chen J., Yu B., Wu X., Pang P., Chen X. (2016). Efficacy of traditional Chinese medicine in treating cancer. Biomed. Rep..

[140] Bigelsen S. (2018). Evidence-based complementary treatment of pancreatic cancer: A review of adjunct therapies including paricalcitol, hydroxychloroquine, intravenous vitamin C, statins, metformin, curcumin, and aspirin. Cancer Manag. Res..

[141] Gao J.J., Song P.P., Qi F.H., Kokudo N., Qu X.J., Tang W. (2012). Evidence-based research on traditional Japanese medicine, Kampo, in treatment of gastrointestinal cancer in Japan. Drug. Discov. Ther..

[142] Valenzuela R., Morales A., Sheen J., Rangel S., Salinas J.J. (2023). The Implementation of Evidence-Based Obesity Education Curricula to Prevent Cancer in a Predominantly Mexican-American Community on the U.S.-Mexico Border. J. Cancer Educ..

[143] Ai A.L., McCormick T.R. (2009). Increasing diversity of Americans’ faiths alongside Baby Boomers’ aging: Implications for chaplain intervention in health settings. J. Health Care Chaplain..

[144] Jaap K., Fluck M., Hunsinger M., Wild J., Arora T., Shabahang M., Blansfield J. (2018). Analyzing the Impact of Compliance with National Guidelines for Pancreatic Cancer Care Using the National Cancer Database. J. Gastrointest. Surg..

[145] Li J.A., Wu W.C., Ji Y., Liu L.X., Rao S.X., Wang D.S., Zhang Y.Q., Yao X.Z., Fan Y., Huang C. (2019). Diagnostic value and patient compliance of a pancreas-oriented multidisciplinary clinic: A retrospective analysis from a Chinese pancreatic disease center. Zhonghua Wai Ke Za Zhi.

[146] Li J.A., Xu Y.L., Ding N., Ji Y., Liu L.X., Rao S.X., Zhang Y.Q., Yao X.Z., Fan Y., Huang C. (2022). Pancreas multidisciplinary team optimizes the diagnosis and treatment of pancreas-related diseases and improves the prognosis of pancreatic cancer patients. Zhonghua Wai Ke Za Zhi.

[147] Sheni R., Qin J., Viswanathan S., Castellucci E., Kalnicki S., Mehta V. (2023). Predictive Factors for Cancer Treatment Delay in a Racially Diverse and Socioeconomically Disadvantaged Urban Population. JCO Oncol. Pract..

[148] Lee H.J., Qian C.L., Landay S.L., O’Callaghan D., Kaslow-Zieve E., Azoba C.C., Fuh C.X., Temel B., Ufere N., Petrillo L.A. (2022). Communicating the Information Needed for Treatment Decision Making among Patients with Pancreatic Cancer Receiving Preoperative Therapy. JCO Oncol. Pract..

[149] Haragi M., Hayakawa M., Watanabe O., Takayama T. (2021). An exploratory study of the efficacy of medical illustration detail for delivering cancer information. J. Vis. Commun. Med..

[150] Santos L.G., Buzdnitskaya T., Rolf B.A., Souza W., Sienko M., Ruiz-Bonilla J.A., Shah B., Jewell P., Jensen L., Horike-Pyne M. (2023). Assessment of a Peer Physician Coaching Partnership between a Designated Cancer Center Genetics Service and a Community Cancer Network Hospital. JAMA Netw. Open.

[151] Wong E.C., Torres V.N., Martinez M.O., Han B., Vue M., Derose K.P. (2023). A parish-based multilevel cluster randomized controlled trial to reduce stigma and mental health treatment disparities among Latino communities. Contemp. Clin. Trials.

[152] Collett L.K., Hudson L., Prichard C., Vanderford N.L. (2023). Using Culturally Focused Storytelling to Empower Appalachian Kentucky Youth to Understand and Address Cancer Disparities in Their Communities. J. Cancer Educ..

[153] Seo J.Y., Park S.H., Choi S.E., Lee M., Strauss S.M. (2023). Development and Modification of a Culturally Tailored Education Program to Prevent Breast Cancer in Korean Immigrant Women in New York City. J. Cancer Educ..

[154] Waterbor J.W., Adams R.M., Robinson J.M., Crabtree F.G., Accortt N.A., Gilliland J. (2004). Disparities between public health educational materials and the scientific evidence that smokeless tobacco use causes cancer. J. Cancer Educ..

[155] El Miedany Y. (2015). MABS: Targeted therapy tailored to the patient’s need. Br. J. Nurs..

[156] Tung H.F., Chen Y.L., Chen C.L., Gee M.J., Muo C.H., Chiu S.L. (2023). How Cultural Behaviors and Superstitions Associate the Willingness to Undergo Cataract Surgery in Taiwan: A Nationwide Survey. Medicina.

[157] Seppala T.T., Burkhart R.A., Katona B.W. (2023). Hereditary colorectal, gastric, and pancreatic cancer: Comprehensive review. BJS Open.

[158] Chapple A., Evans J., McPherson A., Payne S. (2011). Patients with pancreatic cancer and relatives talk about preferred place of death and what influenced their preferences: A qualitative study. BMJ Support. Palliat. Care.

[159] Taylor A.K., Chang D., Chew-Graham C., Rimmer L., Kausar A. (2021). ‘It’s always in the back of my mind’: Understanding the psychological impact of recovery following pancreaticoduodenectomy for cancer: A qualitative study. BMJ Open.

[160] Kummer S., Walter F.M., Chilcot J., Emery J., Sutton S., Scott S.E. (2019). Do cognitive heuristics underpin symptom appraisal for symptoms of cancer?: A secondary qualitative analysis across seven cancers. Psychooncology.

[161] Coleman J., Olsen S.J., Sauter P.K., Baker D., Hodgin M.B., Stanfield C., Emerling A., Hruban R.H., Nolan M.T. (2005). The effect of a Frequently Asked Questions module on a pancreatic cancer Web site patient/family chat room. Cancer Nurs..

[162] Gagliardi A.R., Webster F., Brouwers M.C., Baxter N.N., Finelli A., Gallinger S. (2014). How does context influence collaborative decision-making for health services planning, delivery and evaluation?. BMC Health Serv. Res..

[163] Hurdle V., Ouellet J.F., Dixon E., Howard T.J., Lillemoe K.D., Vollmer C.M., Sutherland F.R., Ball C.G. (2014). Does regional variation impact decision-making in the management and palliation of pancreatic head adenocarcinoma? Results from an international survey. Can. J. Surg..

[164] Mills K., Birt L., Emery J.D., Hall N., Banks J., Johnson M., Lancaster J., Hamilton W., Rubin G.P., Walter F.M. (2017). Understanding symptom appraisal and help-seeking in people with symptoms suggestive of pancreatic cancer: A qualitative study. BMJ Open.

[165] Barello S., Acampora M., Grimaldi L., Maccacaro C., Dell’Acqua S., Spina B., Giangreco D. (2023). “Health without Borders”: Early Findings and Lessons Learned from a Health Promotion Program for Ethnic Minorities Living in Italy. Int. J. Environ. Res. Public Health.

[166] Fernandez-Rozadilla C., Timofeeva M., Chen Z., Law P., Thomas M., Schmit S., Diez-Obrero V., Hsu L., Fernandez-Tajes J., Palles C. (2023). Deciphering colorectal cancer genetics through multi-omic analysis of 100,204 cases and 154,587 controls of European and east Asian ancestries. Nat. Genet..

[167] Odunsi K. (2023). Perspectives on Disparities and Equity in Cancer Outcomes: A Call to Action. Acad. Med..

[168] Acoba J.D., Yin C., Meno M., Abe J., Pagano I., Tamashiro S., Fujinaga K., Braun-Inglis C., Fukui J. (2022). Racial Disparities in Patient-Provider Communication During Telehealth Visits Versus Face-to-face Visits among Asian and Native Hawaiian and Other Pacific Islander Patients with Cancer: Cross-sectional Analysis. JMIR Cancer.

[169] Liu Y., Kornfield R., Yang E.F., Burnside E., Keevil J., Shah D.V. (2022). Patient-provider communication while using a clinical decision support tool: Explaining satisfaction with shared decision making for mammography screening. BMC Med. Inform. Decis. Mak..

[170] Symanski E., An Han H., McCurdy S., Hopkins L., Flores J., Han I., Smith M.A., Caldwell J., Fontenot C., Wyatt B. (2023). Data to Action: Community-Based Participatory Research to Address Concerns about Metal Air Pollution in Overburdened Neighborhoods near Metal Recycling Facilities in Houston. Environ. Health Perspect..

[171] Milton A.J., Flores E.J., Charles E.F., Elezaby M.A., Ward E.C., Lee C.I., Woods R.W., Martin Rother M.D., Strigel R.M., Narayan A.K. (2023). Community-based Participatory Research: A Practical Guide for Radiologists. Radiographics.

[172] Morrell M.A., Willis T.R., Brown D.R., O’Brian C.A., Post S.L., Woloschak G.E., Bonini M.G., Paunesku T., Popovic J., Manning T.M. (2023). Lessons learned in the practice of community-based participatory research with community partner collaboration in study design and implementation: The community scientist model. Cancer Causes Control.

[173] Anderson M.D., Pickner W.J., Begnaud A. (2023). Determinants of Lung Cancer Screening in a Minnesota Urban Indigenous Community: A Community-Based, Participatory, Action-Oriented Study. Cancer Prev. Res..

[174] Rivas G., Rodriguez-Colon S., Ramirez S.I., Galdamez C., Valdez S., Shirley S., Diaz-Myers M., Lengerich E.J. (2023). Evaluation of the Spanish-Language Cancer Educational Webinar Series “Vamos a educarnos contra el cancer” with the RE-AIM Framework. J. Cancer Educ..

[175] Warner Z.C., Gilbert-Gard K., Reid B., Joseph W., Kepka D., Auguste P., Warner E.L. (2023). Knowledge and awareness of colorectal cancer among a predominantly Indigenous Caribbean community. BMC Public Health.

[176] Alpert J.M., Morris B.B., Thomson M.D., Matin K., Brown R.F. (2019). Identifying How Patient Portals Impact Communication in Oncology. Health Commun..

[177] Engle R.L., Bokhour B.G., Rose A.J., Reisman J.I., Jasuja G.K. (2023). Characterizing patient attitudes and beliefs towards testosterone therapy in Veterans Affairs: A qualitative study. Patient Educ. Couns..

[178] Stolwijk M.L., van Nispen R.M.A., van der Ham A.J., Veenman E., van Rens G. (2023). Barriers and facilitators in the referral pathways to low vision services from the perspective of patients and professionals: A qualitative study. BMC Health Serv. Res..

[179] Benham-Hutchins M., Staggers N., Mackert M., Johnson A.H., deBronkart D. (2017). “I want to know everything”: A qualitative study of perspectives from patients with chronic diseases on sharing health information during hospitalization. BMC Health Serv. Res..

[180] Hamilton J.G., Hutson S.P., Frohnmayer A.E., Han P.K., Peters J.A., Carr A.G., Alter B.P. (2015). Genetic Information-Seeking Behaviors and Knowledge among Family Members and Patients with Inherited Bone Marrow Failure Syndromes. J. Genet. Couns..

[181] Ratnapradipa K.L., Ranta J., Napit K., Luma L.B., Robinson T., Dinkel D., Schabloske L., Watanabe-Galloway S. (2022). Qualitative analysis of cancer care experiences among rural cancer survivors and caregivers. J. Rural. Health.

[182] Sanderson P.R., Teufel-Shone N.I., Baldwin J.A., Sandoval N., Robinson F. (2010). Breast cancer education for Navajo women: A pilot study evaluating a culturally relevant video. J. Cancer Educ..

[183] Ye Y. (2014). The role of illness factors and patient satisfaction in using online health support groups. Health Commun..

[184] Bayram T., Sakarya S. (2023). Oppression and internalized oppression as an emerging theme in accessing healthcare: Findings from a qualitative study assessing first-language related barriers among the Kurds in Turkey. Int. J. Equity Health.

[185] Hu S.Y., Reel E., Nisenbaum R., Scheer A.S. (2022). Challenges in Cross-Cultural Communication in Breast Cancer Surgery: Is there a Gender Gap?. J. Cancer Educ..

[186] Rosa W.E., Roberts K.E., Braybrook D., Harding R., Godwin K., Mahoney C., Mathew S., Atkinson T.M., Banerjee S.C., Haviland K. (2023). Palliative and end-of-life care needs, experiences, and preferences of LGBTQ+ individuals with serious illness: A systematic mixed-methods review. Palliat. Med..

[187] Morena N., Zelt N., Nguyen D., Dionne E., Rentschler C.A., Greyson D., Meguerditchian A.N. (2023). The Use of Web-Based Patient Reviews to Assess Medical Oncologists’ Competency: Mixed Methods Sequential Explanatory Study. JMIR Form. Res..

[188] Stewart E.C., Davis J.S., Walters T.S., Chen Z., Miller S.T., Duke J.M., Alexander L.R., Akohoue S.A., Russell R., Rowan N. (2023). Development of strategies for community engaged research dissemination by basic scientists: A case study. Transl. Res..

[189] Broadbridge E., Greene K., Venetis M.K., Lee L.E., Banerjee S.C., Saraiya B., Devine K.A. (2023). Facilitating psychological adjustment for breast cancer patients through empathic communication and uncertainty reduction. Patient Educ. Couns..

[190] Korsvold L., Mellblom A.V., Lie H.C., Ruud E., Loge J.H., Finset A. (2016). Patient-provider communication about the emotional cues and concerns of adolescent and young adult patients and their family members when receiving a diagnosis of cancer. Patient Educ. Couns..

[191] Posma E.R., van Weert J.C., Jansen J., Bensing J.M. (2009). Older cancer patients’ information and support needs surrounding treatment: An evaluation through the eyes of patients, relatives and professionals. BMC Nurs..

[192] Lin J.J., Lake J., Wall M.M., Berman A.R., Salazar-Schicchi J., Powell C., Keller S.M., Halm E.A., Leventhal H., Wisnivesky J.P. (2014). Association of patient-provider communication domains with lung cancer treatment. J. Thorac. Oncol..

[193] Makoul G. (2003). The interplay between education and research about patient-provider communication. Patient Educ. Couns..

[194] Bogdan-Lovis E., Zhuang J., Goldbort J., Shareef S., Bresnahan M., Kelly-Blake K., Elam K. (2023). Do Black birthing persons prefer a Black health care provider during birth? Race concordance in birth. Birth.

[195] Chou W.Y., Han P., Pilsner A., Coa K., Greenberg L., Blatt B. (2011). Interdisciplinary research on patient-provider communication: A cross-method comparison. Commun. Med..

[196] Seible D.M., Kundu S., Azuara A., Cherry D.R., Arias S., Nalawade V.V., Cruz J., Arreola R., Martinez M.E., Nodora J.N. (2021). The Influence of Patient-Provider Language Concordance in Cancer Care: Results of the Hispanic Outcomes by Language Approach (HOLA) Randomized Trial. Int. J. Radiat. Oncol. Biol. Phys..

[197] Shaw A.C., McQuade J.L., Reilley M.J., Nixon B., Baile W.F., Epner D.E. (2019). Integrating Storytelling into a Communication Skills Teaching Program for Medical Oncology Fellows. J. Cancer Educ..

[198] Ward M., Elder B., Habtemariam M. (2021). Current Testing Guidelines: A Retrospective Analysis of a Community-Based Hereditary Cancer Program. J. Adv. Pract. Oncol..

[199] Eliacin J., Burgess D., Rollins A.L., Patterson S., Damush T., Bair M.J., Salyers M.P., Spoont M., Chinman M., Slaven J.E. (2023). Outcomes of a peer-led navigation program, PARTNER-MH, for racially minoritized Veterans receiving mental health services: A pilot randomized controlled trial to assess feasibility and acceptability. Transl. Behav. Med..

[200] Gage-Bouchard E.A. (2017). Social support, flexible resources, and health care navigation. Soc. Sci. Med..

[201] Molina Y., Kim S.J., Berrios N., Glassgow A.E., San Miguel Y., Darnell J.S., Pauls H., Vijayasiri G., Warnecke R.B., Calhoun E.A. (2018). Patient Navigation Improves Subsequent Breast Cancer Screening after a Noncancerous Result: Evidence from the Patient Navigation in Medically Underserved Areas Study. J. Womens Health.

[202] Molina Y., Glassgow A.E., Kim S.J., Berrios N.M., Pauls H., Watson K.S., Darnell J.S., Calhoun E.A. (2017). Patient Navigation in Medically Underserved Areas study design: A trial with implications for efficacy, effect modification, and full continuum assessment. Contemp. Clin. Trials.

[203] Heisler M., Lapidos A., Henderson J., Guzman R.M., Wolfe J., Meyer P., Law D., Kieffer E.C., Ernst C., Djelaj V. (2019). Study protocol for a Community Health Worker (CHW)-led comprehensive neighborhood-focused program for medicaid enrollees in detroit. Contemp. Clin. Trials Commun..

[204] Izumi B.T., Schulz A.J., Israel B.A., Reyes A.G., Martin J., Lichtenstein R.L., Wilson C., Sand S.L. (2010). The one-pager: A practical policy advocacy tool for translating community-based participatory research into action. Prog. Community Health Partnersh..

[205] Pulte D., Redaniel M.T., Brenner H., Jeffreys M. (2012). Changes in survival by ethnicity of patients with cancer between 1992-1996 and 2002–2006: Is the discrepancy decreasing?. Ann. Oncol..

[206] Yang H.T., Wu M.C., Shun S.C. (2018). Care Plan for Resuming the Physical Activity of Patients with Pancreatic Cancer and Diabetes after Surgery. Hu Li Za Zhi.

[207] Arthur A.E., Delk A., Demark-Wahnefried W., Christein J.D., Contreras C., Posey J.A., Vickers S., Oster R., Rogers L.Q. (2016). Pancreatic cancer survivors’ preferences, barriers, and facilitators related to physical activity and diet interventions. J. Cancer Surviv..

[208] Youn P., Li H., Milano M.T., Stovall M., Constine L.S., Travis L.B. (2013). Long-term survival among Hodgkin’s lymphoma patients with gastrointestinal cancer: A population-based study. Ann Oncol.

[209] Huang B.Z., Wang S., Bogumil D., Wilkens L.R., Wu L., Blot W.J., Zheng W., Shu X.O., Pandol S.J., Le Marchand L. (2021). Red meat consumption, cooking mutagens, NAT1/2 genotypes and pancreatic cancer risk in two ethnically diverse prospective cohorts. Int. J. Cancer.

[210] Zoellner J.M., Porter K.J., Brock D.P., Mitchell E.M.K., Chapman H., Clarkston D., Cohn W., Hauser L., Morris D.W., Ramey S.Y. (2021). Advancing engagement and capacity for rural cancer control: A mixed-methods case study of a Community-Academic Advisory Board in the Appalachia region of Southwest Virginia. Res. Involv. Engagem..

[211] Batai K., Sanderson P.R., Joshweseoma L., Burhansstipanov L., Russell D., Joshweseoma L., Hsu C.H. (2022). Formative Assessment to Improve Cancer Screenings in American Indian Men: Native Patient Navigator and mHealth Texting. Int. J. Environ. Res. Public Health.

[212] Dockery L.E., Motwani A., Ding K., Doescher M., Dvorak J.D., Moore K.N., Holman L.L. (2018). Improving cancer care for American Indians with cervical cancer in the Indian Health Service (IHS) system-Navigation may not be enough. Gynecol. Oncol..

[213] Bernardes C.M., Martin J., Cole P., Kitchener T., Cowburn G., Garvey G., Walpole E., Valery P.C. (2018). Lessons learned from a pilot study of an Indigenous patient navigator intervention in Queensland, Australia. Eur. J. Cancer Care.

[214] Burhansstipanov L., Krebs L.U., Dignan M.B., Jones K., Harjo L.D., Watanabe-Galloway S., Petereit D.G., Pingatore N.L., Isham D. (2014). Findings from the native navigators and the Cancer Continuum (NNACC) study. J. Cancer Educ..

[215] Arredondo E.M., Haughton J., Ayala G.X., Slymen D., Sallis J.F., Perez L.G., Serrano N., Ryan S., Valdivia R., Lopez N.V. (2022). Two-year outcomes of Faith in Action/Fe en Accion: A randomized controlled trial of physical activity promotion in Latinas. Int. J. Behav. Nutr. Phys. Act..

[216] Lee S., Niakosari Hadidi N., Lindgren B.R., Kelley R., Lindquist R. (2022). Peer Group Support Intervention to Reduce Cardiovascular Disease Risk for African American Men According to Life’s Simple 7 in Faith-Based Communities. Res. Theory Nurs. Pract..

[217] Knott C.L., Miech E.J., Slade J., Woodard N., Robinson-Shaneman B.J., Huq M. (2022). Evaluation of organizational capacity in the implementation of a church-based cancer education program. Glob. Implement. Res. Appl..

[218] Maxwell A.E., Lucas-Wright A., Gatson J., Cindy Chang L., Crespi C.M. (2020). Training Community Health Advisors in African American Churches: Do Training Outcomes Predict Performance?. J. Cancer Educ..

[219] Hamdiui N., Bouman M.P.A., Stein M.L., Crutzen R., Keskin D., Afrian A., van Steenbergen J.E., van den Muijsenbergh M., Timen A. (2022). The development of a culturally sensitive educational video: How to facilitate informed decisions on cervical cancer screening among Turkish- and Moroccan-Dutch women. Health Expect.

[220] Del Valle D.D., Pardo J.A., Maselli A.M., Valero M.G., Fan B., Seyidova N., James T.A., Lee B.T. (2021). Evaluation of online Spanish and English health materials for preventive mastectomy. are we providing adequate information?. Breast Cancer Res. Treat..

[221] Saif M.W. (2021). From Screening to Treatment of Pancreatic Cancer: A Comprehensive Review. JOP.

[222] Dionne-Odom J.N., Ejem D., Azuero A., Taylor R.A., Rocque G.B., Turkman Y., Thompson M.A., Knight S.J., Martin M.Y., Bakitas M.A. (2018). Factors Associated with Family Caregivers’ Confidence in Future Surrogate Decision Making for Persons with Cancer. J. Palliat. Med..

[223] Tang C.C., Draucker C., Tejani M.A., Von Ah D. (2018). Patterns of interactions among patients with advanced pancreatic cancer, their caregivers, and healthcare providers during symptom discussions. Support Care Cancer.

[224] Sharp L., Mentor K., Deane J., Watson E., Roberts K.J., Silva M., Phillips M., Siriwardena A.K., Hammond J., Bradshaw A. (2023). Assessing impact, needs and quality-of-life among informal carers of people with pancreatic cancer, a prospective study: The PAN-CARER study protocol. BMJ Open.

[225] Abma I.L., Roelofs L.C.G., van der Kolk M.B., Mulder S.F., Schers H.J., Hermens R., van der Wees P.J. (2022). Roles of general practitioners in shared decision-making for patients with cancer: A qualitative study. Eur. J. Cancer Care.

[226] Griffioen I.P.M., Rietjens J.A.C., Melles M., Snelders D., Homs M.Y.V., van Eijck C.H., Stiggelbout A.M. (2021). The bigger picture of shared decision making: A service design perspective using the care path of locally advanced pancreatic cancer as a case. Cancer Med..

[227] Reissig T.M., Tzianopoulos I., Liffers S.T., Rosery V.K., Guyot M., Ting S., Wiesweg M., Kasper S., Meister P., Herold T. (2023). Smaller panel, similar results: Genomic profiling and molecularly informed therapy in pancreatic cancer. ESMO Open.

[228] Kim C.A., Lelond S., Daeninck P.J., Rabbani R., Lix L., McClement S., Chochinov H.M., Goldenberg B.A. (2023). The impact of early palliative care on the quality of life of patients with advanced pancreatic cancer: The IMPERATIVE case-crossover study. Support Care Cancer.

[229] Jardim S.R., de Souza L.M.P., de Souza H.S.P. (2023). The Rise of Gastrointestinal Cancers as a Global Phenomenon: Unhealthy Behavior or Progress?. Int. J. Environ. Res. Public Health.

[230] El Kaoutari A., Fraunhoffer N.A., Audebert S., Camoin L., Berthois Y., Gayet O., Roques J., Bigonnet M., Bongrain C., Ciccolini J. (2023). Pancreatic ductal adenocarcinoma ubiquitination profiling reveals specific prognostic and theranostic markers. EBioMedicine.

[231] Dhani H., Hinestrosa J.P., Izaguirre-Carbonell J., Balcer H.I., Kurzrock R., Billings P.R. (2023). Case Report: Early detection of pancreatic pre-cancer lesion in multimodal approach with exosome liquid biopsy. Front. Oncol..

[232] Zhao Y., Tang J., Jiang K., Liu S.Y., Aicher A., Heeschen C. (2023). Liquid biopsy in pancreatic cancer-Current perspective and future outlook. Biochim. Biophys. Acta Rev. Cancer.

[233] Roth G.S., Fayet Y., Benmameche-Medjahed S., Ducimetiere F., Charreton A., Cropet C., Chabaud S., Marion-Audibert A.M., Berthelet O., Walter T. (2022). Structural and Socio-Spatial Determinants Influencing Care and Survival of Patients with a Pancreatic Adenocarcinoma: Results of the PANDAURA Cohort. Cancers.

[234] Wang F., Shu X., Pal T., Berlin J., Nguyen S.M., Zheng W., Bailey C.E., Shu X.O. (2022). Racial/Ethnic Disparities in Mortality Related to Access to Care for Major Cancers in the United States. Cancers.

[235] Raoof S., Lee R.J., Jajoo K., Mancias J.D., Rebbeck T.R., Skates S.J. (2022). Multicancer Early Detection Technologies: A Review Informed by Past Cancer Screening Studies. Cancer Epidemiol. Biomark. Prev..

[236] Bledsoe M.J., Grizzle W.E. (2013). Use of human specimens in research: The evolving United States regulatory, policy, and scientific landscape. Diagn. Histopathol..

[237] Howell L.A., Sinicrope P.S., Brockman T.A., Patten C.A., Decker P.A., Ehlers S.L., Nadeau A., Rabe K.G., Breitkopf C.R., Petersen G.M. (2013). Receptivity and preferences of pancreatic cancer family members for participating in lifestyle programs to reduce cancer risk. Hered. Cancer Clin. Pract..

[238] Lavinia Loeffler C.M., El Nahhas O.S.M., Muti H.S., Seibel T., Cifci D., van Treeck M., Gustav M., Carrero Z.I., Gaisa N.T., Lehmann K.V. (2023). Direct prediction of Homologous Recombination Deficiency from routine histology in ten different tumor types with attention-based Multiple Instance Learning: A development and validation study. medRxiv.

[239] Guest D.D., Cox T., Voss A.C., Kelley K., Ma X., Nguyen A., McMillen K., Williams V., Lee J.A., Petersen J. (2023). Assessing Impact of Nutrition Care by Registered Dietitian Nutritionists on Patient Medical and Treatment Outcomes in Outpatient Cancer Clinics: A Cohort Feasibility Study. Nutr. Cancer.

[240] Pedrosa L., Araujo I.K., Cuatrecasas M., Soy G., Lopez S., Maurel J., Sanchez-Montes C., Montironi C., Sauri T., Sendino O. (2023). Targeted transcriptomic analysis of pancreatic adenocarcinoma in EUS-FNA samples by NanoString technology. Front. Mol. Biosci..

[241] Tsai Y.S., Chareddy Y.S., Price B.A., Parker J.S., Pecot C.V. (2023). An integrated model for predicting KRAS dependency. PLoS Comput. Biol..

[242] Yang H., Li W., Ren L., Yang Y., Zhang Y., Ge B., Li S., Zheng X., Liu J., Zhang S. (2023). Progress on diagnostic and prognostic markers of pancreatic cancer. Oncol. Res..

[243] Yu Z., Yang Y., Fang W., Hu P., Liu Y., Shi J. (2023). Dual Tumor Exosome Biomarker Co-recognitions Based Nanoliquid Biopsy for the Accurate Early Diagnosis of Pancreatic Cancer. ACS Nano..

[244] Heiselman J.S., Ecker B.L., Langdon-Embry L., O’Reilly E.M., Miga M.I., Jarnagin W.R., Do R.K.G., Horvat N., Wei A.C., Chakraborty J. (2023). Registration-based biomarkers for neoadjuvant treatment response of pancreatic cancer via longitudinal image registration. J. Med. Imaging.

[245] Vannini I., Rossi T., Melloni M., Valgiusti M., Urbini M., Passardi A., Bartolini G., Gallio C., Azzali I., Bandini S. (2023). Analysis of EVs from patients with advanced pancreatic cancer identifies antigens and miRNAs with predictive value. Mol. Ther. Methods Clin. Dev..

[246] Kane L.E., Mellotte G.S., Conlon K.C., Ryan B.M., Maher S.G. (2021). Multi-Omic Biomarkers as Potential Tools for the Characterisation of Pancreatic Cystic Lesions and Cancer: Innovative Patient Data Integration. Cancers.

[247] Turanli B., Yildirim E., Gulfidan G., Arga K.Y., Sinha R. (2021). Current State of “Omics” Biomarkers in Pancreatic Cancer. J. Pers. Med..

[248] Xu D., Wang Y., Liu X., Zhou K., Wu J., Chen J., Chen C., Chen L., Zheng J. (2021). Development and clinical validation of a novel 9-gene prognostic model based on multi-omics in pancreatic adenocarcinoma. Pharmacol. Res..

[249] De Pastena M., Esposito A., Paiella S., Surci N., Montagnini G., Marchegiani G., Malleo G., Secchettin E., Casetti L., Ricci C. (2021). Cost-effectiveness and quality of life analysis of laparoscopic and robotic distal pancreatectomy: A propensity score-matched study. Surg. Endosc..

[250] Chen Q., Merath K., Bagante F., Akgul O., Dillhoff M., Cloyd J., Pawlik T.M. (2018). A Comparison of Open and Minimally Invasive Surgery for Hepatic and Pancreatic Resections among the Medicare Population. J. Gastrointest. Surg..

[251] Reddy A., Amarnani A., Chen M., Dynes S., Flores B., Moshchinsky A., Lee Y.J., Kurbatov V., Shapira I., Vignesh S. (2020). Privacy Concerns about Personal Health Information and Fear of Unintended Use of Biospecimens Impact Donations by African American Patients. J. Cancer Educ..

[252] Peota C. (2012). A question of consent. Minn. Med..

[253] Portman D.G., Thirlwell S., Donovan K.A., Ellington L. (2021). Virtual Teaming: Leveraging Team Science Sense-Making During COVID-19. J. Patient Exp..

[254] Katz M.H., Slack R., Bruno M., McMillan J., Fleming J.B., Lee J.E., Bednarski B., Papadopoulos J., Matin S.F. (2016). Outpatient virtual clinical encounters after complex surgery for cancer: A prospective pilot study of “TeleDischarge”. J. Surg. Res..

[255] Satturwar S., Monaco S.E., Xing J., Pantanowitz L. (2020). The utility of cell blocks for international cytopathology teleconsultation by whole slide imaging. Cytopathology.

[256] Gustavell T., Sundberg K., Langius-Eklof A. (2020). Using an Interactive App for Symptom Reporting and Management Following Pancreatic Cancer Surgery to Facilitate Person-Centered Care: Descriptive Study. JMIR Mhealth Uhealth.

[257] Gustavell T., Sundberg K., Segersvard R., Wengstrom Y., Langius-Eklof A. (2019). Decreased symptom burden following surgery due to support from an interactive app for symptom management for patients with pancreatic and periampullary cancer. Acta Oncol..

[258] Jiang J., Chao W.L., Culp S., Krishna S.G. (2023). Artificial Intelligence in the Diagnosis and Treatment of Pancreatic Cystic Lesions and Adenocarcinoma. Cancers.

[259] Tovar D.R., Rosenthal M.H., Maitra A., Koay E.J. (2023). Potential of artificial intelligence in the risk stratification for and early detection of pancreatic cancer. Artif. Intell. Surg..

[260] Gagliardi A.R., Soong D., Gallinger S. (2016). Identifying Factors Influencing Pancreatic Cancer Management to Inform Quality Improvement Efforts and Future Research: A Scoping Systematic Review. Pancreas.

[261] Low C.A., Li M., Vega J., Durica K.C., Ferreira D., Tam V., Hogg M., Zeh Iii H., Doryab A., Dey A.K. (2021). Digital Biomarkers of Symptom Burden Self-Reported by Perioperative Patients Undergoing Pancreatic Surgery: Prospective Longitudinal Study. JMIR Cancer.

[262] Cos H., Li D., Williams G., Chininis J., Dai R., Zhang J., Srivastava R., Raper L., Sanford D., Hawkins W. (2021). Predicting Outcomes in Patients Undergoing Pancreatectomy Using Wearable Technology and Machine Learning: Prospective Cohort Study. J. Med. Internet Res..

[263] Nipp R.D., Gaufberg E., Vyas C., Azoba C., Qian C.L., Jaggers J., Weekes C.D., Allen J.N., Roeland E.J., Parikh A.R. (2022). Supportive Oncology Care at Home Intervention for Patients with Pancreatic Cancer. JCO Oncol. Pract..

